# Hole-Transporting Materials for Perovskite Solar Cells Employing an Anthradithiophene Core

**DOI:** 10.1021/acsami.1c05890

**Published:** 2021-06-09

**Authors:** José Santos, Joaquín Calbo, Rafael Sandoval-Torrientes, Inés García-Benito, Hiroyuki Kanda, Iwan Zimmermann, Juan Aragó, Mohammad Khaja Nazeeruddin, Enrique Ortí, Nazario Martín

**Affiliations:** † Facultad de Ciencias Químicas, Universidad Complutense de Madrid, Madrid 28040, Spain; ‡ Instituto de Ciencia Molecular, Universidad de Valencia, Paterna 46980, Spain; § Group for Molecular Engineering of Functional Materials, EPFL VALAIS, Sion 1951, Switzerland; ∥ IMDEA-Nanociencia, Ciudad Universitaria de Cantoblanco, Madrid 28049, Spain

**Keywords:** anthradithiophene, hole-transporting material, perovskite, solar cells, theoretical calculations

## Abstract

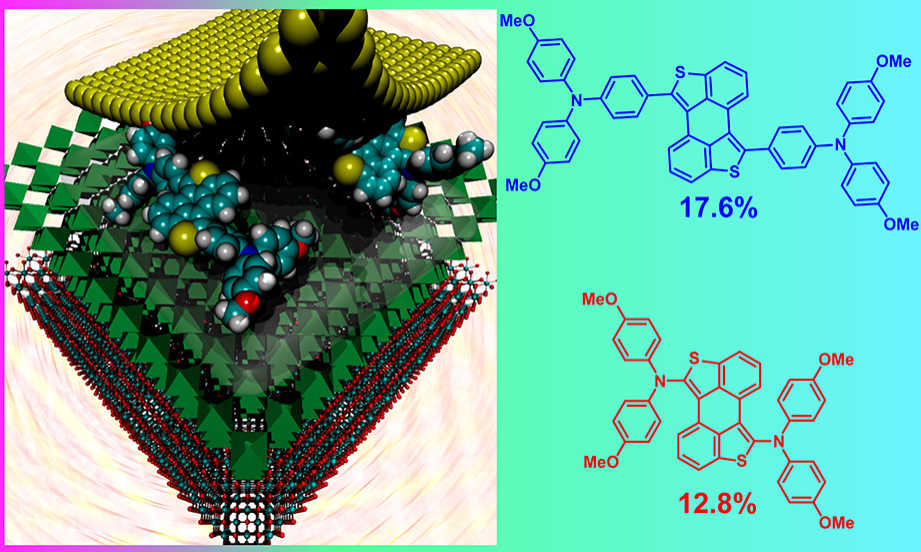

A decade after the report of the first efficient perovskite-based solar cell, development of novel hole-transporting materials (HTMs) is still one of the main topics in this research field. Two of the main advance vectors of this topic lie in obtaining materials with enhanced hole-extracting capability and in easing their synthetic cost. The use of anthra[1,9-*bc*:5,10-*b*′*c*′]dithiophene (ADT) as a flat π-conjugated frame for bearing arylamine electroactive moieties allows obtaining two novel highly efficient HTMs from very cheap precursors. The solar cells fabricated making use of the mixed composition (FAPbI_3_)_0.85_(MAPbBr_3_)_0.15_ perovskite and the novel ADT-based HTMs show power conversion efficiencies up to 17.6% under 1 sun illumination compared to the 18.1% observed when using the benchmark compound 2,2′,7,7′-tetrakis(*N*,*N*-di-*p*-methoxyphenylamine)-9,9′-spirobifluorene (spiro-OMeTAD). Detailed density functional theory calculations allow rationalization of the observed opto-electrochemical properties and predict a flat molecular structure with a low reorganization energy that supports the high conductivity measured for the best-performing HTM.

## Introduction

The relevance of the research on perovskites for solar energy conversion can be weighted in terms of the publication volume on the topic. The astonishing number of 15,996 scientific articles referring to “perovskites for photovoltaics” in the 2009–2021 period (more than 3,600 articles in 2019), with Miyasaka’s paper being cited more than 9,800 times,^1^ perfectly reflects how hot this topic is. The research effort these numbers represent translates into the power conversion efficiency (PCE) leap reported for perovskite-based solar cells from 3.8% in 2009 to the current record of 25.5%.^2^ Originally consisting of an active layer of the CH_3_NH_3_PbI_3_ hybrid perovskite deposited on top of a layer of mesoporous TiO_2_, perovskite solar cells (PSCs) have undergone many modifications. Sandwiching the perovskite active layer between an n-type and a p-type semiconducting material, respectively acting as electron- and hole-transporting layers, allowed a major breakthrough reaching 9.7% PCE when employing spiro-OMeTAD (2,2′,7,7′-tetrakis(*N*,*N*-di-*p*-methoxyphenylamine)-9,9′-spirobifluorene) as the hole-transporting material (HTM).^3^ This improvement marked the development of new organic small-molecule HTMs as one of the main research lines on PSCs.^4−13^ A second research line to improve the performance and stability of PSCs consists of researching new perovskite formulations by modifying both the cations and anions of the ABX_3_ stoichiometry. Such modifications generally lead to perovskites with improved characteristics such as reduced band gap and enhanced electronic properties as when substituting Pb^2+^ by the more environmentally friendly Sn^2+^. Replacement of the organic methylammonium (MA) cation by Cs^+^ or formamidinium (FA)^14,15^ or the halides (Cl^–^ or Br^–^ for I^–^) influences the stability, absorption, and electronic behavior.^1,16,17^ Formulations combining these modifications provide mixed compositional perovskites such as Cs_*x*_(MA_0.17_FA_0.83_)_(100–*x*)_Pb(I_0.83_Br_0.17_)_3_, MAPb(I_1–*x*_Br_*x*_)_3_, or [FAPbI_3_]_0.85_[MAPbBr_3_]_0.15_ with increased stability and efficiencies up to 22%.^18−20^ More recently, increased attention is being devoted to the development of the electron-transporting layer^21^ by introducing fullerene derivatives,^22−25^ azaacenes,^26,27^ and perylene diimides,^28,29^ among others, as n-type materials for replacing the typical inorganic oxide layer (TiO_2_, ZnO, SnO_2_, etc.).^30−32^ Another approach to the PSC research is the study of the effect of introducing certain additives to the active layer, which has proved very successful in improving perovskite’s efficiency,^33^ long-term stability,^34,35^ water resistance,^16,36^ and stability against air.^37^

**Chart 1:**
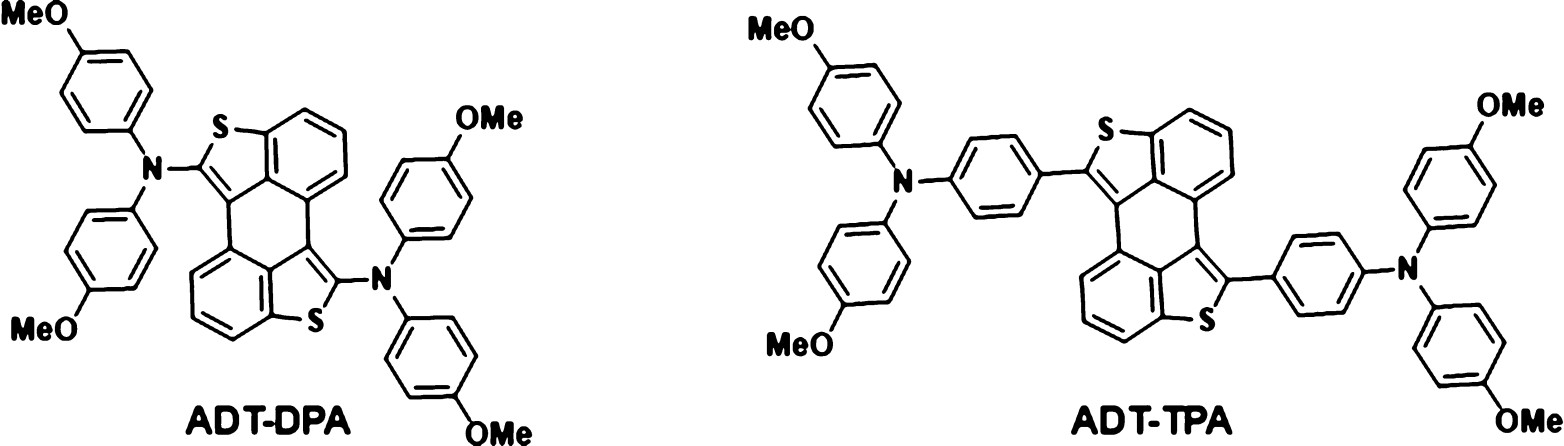
Chemical Structures of the Novel **ADT-DPA** and **ADT-TPA** HTMs

The two main design rules an efficient HTM must meet are (1) having the highest-occupied molecular orbital (HOMO) energetically located in between the valence band edge of the active layer and the cathode’s work function and (2) showing high hole conductivity and mobility. Meeting these two requisites ensures the efficient extraction of holes from the device. The last requirement is strongly dependent on the crystalline structure and the morphology of the HTM, properties which cannot, unfortunately, be predicted. Molecules bearing arylamines as electroactive functionalities have demonstrated to successfully meet the energetic alignment requisite when using perovskites as the active layer.^3−11,38−40^ In this sense, spiro-OMeTAD is the benchmark compound to which all HTMs are compared with due to its good performance in terms of efficiency, reproducibility, and stability. However, the high cost associated with its synthesis and purification stimulates chemists to develop new cheaper and more efficient HTMs. In this publication, we present two novel HTMs consisting of an anthra[1,9-*bc*:5,10-*b*′*c*′]dithiophene (ADT) central core, showcasing two arylamine electroactive moieties appended to its frame, as depicted in Chart 1. Compared to the synthesis of the spiro-OMeTAD framework, the synthesis of ADT is a high-yield, three-step procedure from inexpensive starting materials. The PSCs fabricated employing **ADT-DPA** and **ADT-TPA** show moderate to high efficiencies, in the case of **ADT-TPA** being very similar to that of the spiro-OMeTAD reference.

## Results and Discussion

### Materials Synthesis and Characterization

**Figure 1:**
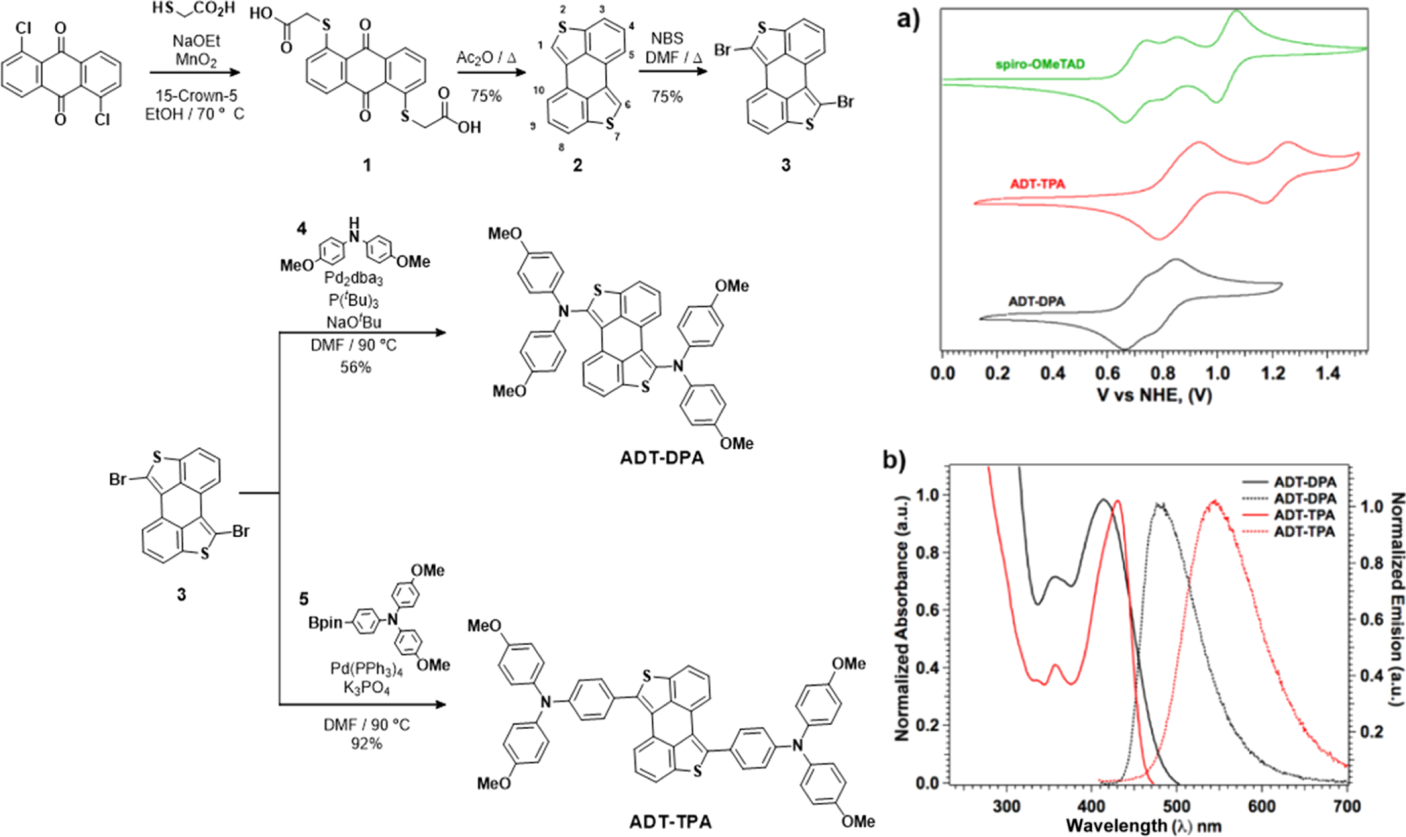
Synthetic route to the new HTMs (left). (a) Cyclic voltammograms of **ADT-DPA** and **ADT-TPA** along with spiro-OMeTAD recorded in the CH_2_Cl_2_ solution at a scan rate of 100 mV s^–1^. (b) Normalized absorption and emission spectra of **ADT-DPA** and **ADT-TPA** recorded in the CH_2_Cl_2_ degassed solution.

The synthesis of ADT was first reported by Wudl et al. in 1979 in a moderate to low yield (43%).^41^ However, the improved method described elsewhere^42^ allows achieving higher yields (75%). Thiolation of 1,5-dichloroanthracene-9,10-dione with mercaptoacetic acid in the presence of sodium ethoxide, manganese(II) oxide, and 15-crown-ether yields dicarboxylate anthraquinone **1** as an insoluble dark solid. Treatment of the suspension of **1** in refluxing acetic anhydride promotes intramolecular cyclization, providing anthradithiophene (**2**) in good yield. Bromination employing *N*-bromosuccinimide in hot dimethylformamide allows functionalizing positions 1 and 6 of the ADT molecule (**3**). Target molecules **ADT-DPA** and **ADT-TPA** are obtained by the corresponding palladium-mediated cross-coupling (Figure 1; full synthetic details and characterization may be found in the Supporting Information). Both novel HTMs show good thermal properties and stability, decomposing over 387 and 327 °C for **ADT-TPA** and **ADT-DPA**, respectively (Figure S1a). Differential scanning calorimetry measurements of **ADT-TPA** show melting point at 250.1 °C and glass transition at 131.7 °C for the second heating scan and 134.9 °C for the third. In the case of **ADT-DPA**, a melting point at 262.1 °C is found, along with a glass transition at 116.7 °C for the second heating scan and 119.4 °C for the third (Figure S1b).

To gain an insight into the structural and electronic properties of the **ADT-DPA** and **ADT-TPA** HTMs, density functional theory (DFT) calculations were carried out at the B3LYP/6-31G** level in the presence of CH_2_Cl_2_ as the solvent. The pendant DPA and TPA moieties, the ADT core, and the reference spiro-OMeTAD compound were also calculated for comparison purposes (see the Supporting Information for full computational details). The structures calculated for the HTMs show a very flat arrangement for **ADT-TPA**, with both TPA units lying almost in-plane with the ADT core, whereas the DPA moieties in **ADT-DPA** are almost orthogonal to the central ADT core (Figure S2). The analysis of the bond lengths computed for the ADT core (Figure S3) evidences the loss of the anthracene-like structural pattern: the external benzenes exhibit a delocalized aromatic structure with minimal C–C bond-order alternation (lengths in the 1.395–1.415 Å range), whereas the central benzene ring presents a more localized structure and acts as a bridge between the external rings with C–C distances of 1.448 and 1.466 Å. On the other hand, the fused thiophene rings preserve their characteristic single-double C–C bond-length alternation, with the edge C–C double bond length being 1.367 Å and the single and double bonds shared with the anthracene being 1.448 and 1.407 Å, respectively. Therefore, the ADT core can be assimilated to two benzothiophene units connected by single C–C bonds (1.466 Å). For the **ADT-DPA** and **ADT-TPA** molecules, the anchoring of the two DPA or TPA units hardly causes any substantial change in the bond lengths of the conjugated core, with the exception of the C–C and C–S thiophene bonds to which the DPA/TPA units are directly attached (Figure S3).

The electrochemical behavior of the new HTMs was studied by cyclic voltammetry measurements. As expected from the electron-rich nature of the arylamine moieties, both molecules show anodic activity (see Figure 1a). Compound **ADT-TPA** exhibits two well-defined oxidation waves (E_1/2_^ox,1^ = 0.93; E_1/2_^ox,2^ = 1.25 V *vs*. NHE), which contrasts with derivative **ADT-DPA**, showing a broad oxidation process comprising two waves at a sensibly lower potential (E_1/2_^ox^ = 0.74 V). HOMO energy levels, estimated from the first oxidation potential, of −5.18 and −5.37 eV for **ADT-DPA** and **ADT-TPA**, respectively, are found in Table 1, which are similar to the value recorded for spiro-OMeTAD (−5.16 eV).^43^ The slightly deeper HOMO of derivative **ADT-TPA** allows a closer energy alignment with the valence band of the perovskite (ca. −5.65 eV), in turn yielding a higher open circuit potential.

**Table 1:** Electrochemical and Photophysical Parameters of HTMs **ADT-DPA** and **ADT-TPA**

HTM	*E*_1/2_^ox^ [V]^a^	*E*_HOMO_ [eV]^b^	λ_max_^abs^ [nm]	λ_max_^em^ [nm]	*E*_0–0_ [eV]^c^	*E*_LUMO_ [eV]^d^
**ADT-DPA**	0.74	–5.18	413	476	2.73	–2.45
**ADT-TPA**	0.93	–5.37	431	547	2.65	–2.72

a Recorded *versus* normal hydrogen electrode (NHE).

b *E*_HOMO_ estimated as *E*_HOMO_ = −4.44 eV – E_1/2_^ox^.

c Optical band gap estimated from the intersection of the absorption and emission spectra.

d *E*_LUMO_ estimated as *E*_LUMO_ = *E*_HOMO_ + *E*_0–0_.

**Figure 2:**
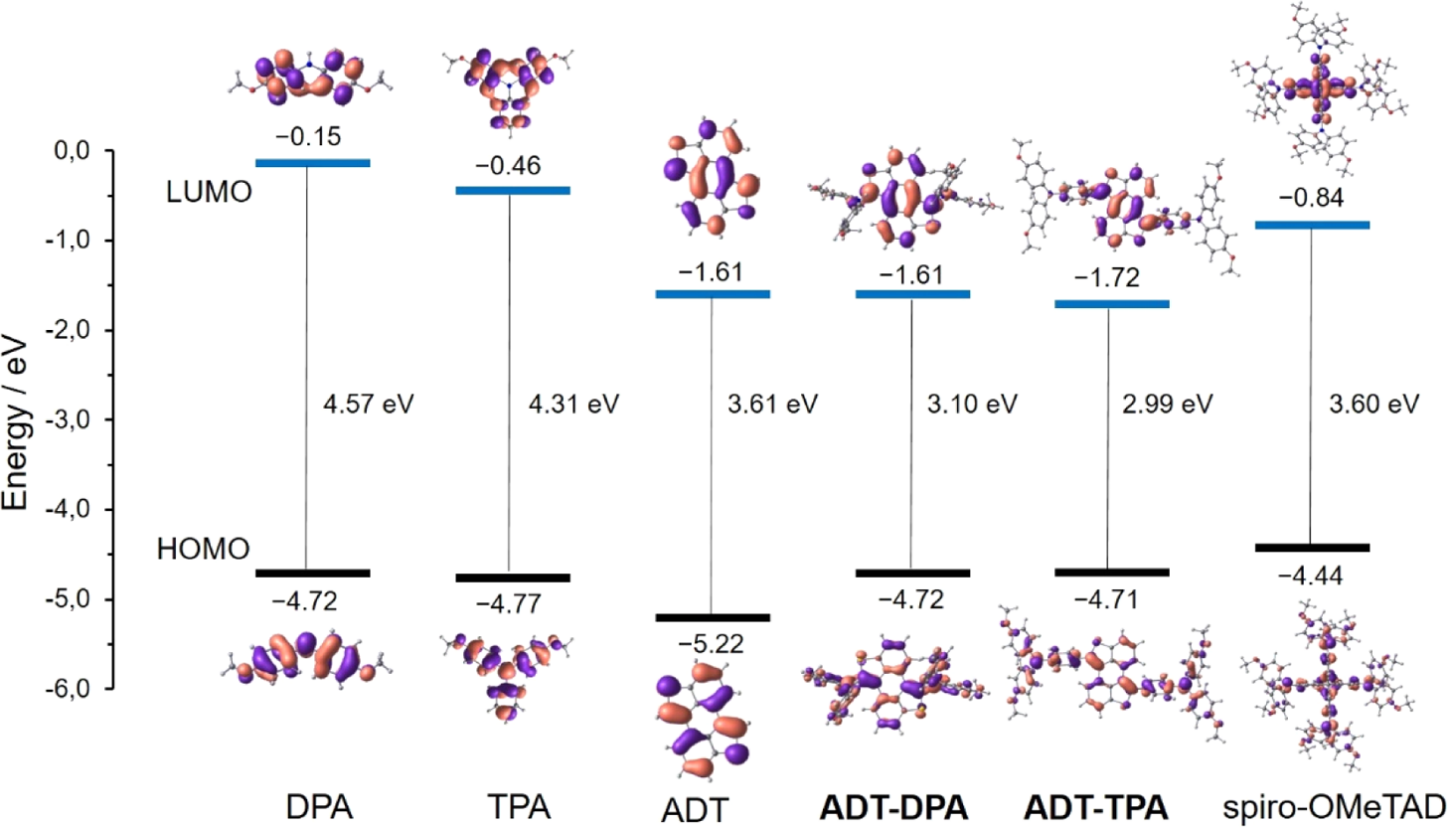
Energy diagram displaying the frontier molecular orbitals computed at the B3LYP/6-31G** level in CH_2_Cl_2_ for the DPA and TPA units, the ADT core, the **ADT-DPA** and **ADT-TPA** HTMs, and the spiro-OMeTAD reference compound.

Figure 2 displays the frontier molecular orbitals calculated at the B3LYP/6-31G** level for the HTMs, their constituent fragments, and the reference compound spiro-OMeTAD. The HOMO of the ADT core (−5.22 eV) is significantly lower in energy than the HOMO levels computed for the DPA and TPA arylamines (−4.72 and −4.77 eV, respectively) in line with the weaker electron-donating character of the ADT moiety. The HOMOs of **ADT-DPA** (−4.72 eV) and **ADT-TPA** (−4.71 eV) retain almost the same energy as the parent arylamines, although spreading all over the whole molecules. In contrast, the lowest-unoccupied molecular orbital (LUMO) is mainly located over the ADT core for **ADT-DPA** (−1.61 eV) and slightly comprises the first neighboring phenyl ring of the TPA moieties in **ADT-TPA** (−1.72 eV). The energies calculated for the HOMO are close to that calculated for the reference spiro-OMeTAD (−4.44 eV). It is of note that an important and opposite charge transfer takes place from the peripheral DPA and TPA units to the sulfur-rich central core. **ADT-DPA** holds a total net charge of 0.21e in its core, whereas a charge of −0.25e is predicted for the core of **ADT-TPA**. These compounds are therefore significantly polarized.

To gain a better insight into the oxidation process, the oxidized species (cations and dications) of **ADT-DPA** and **ADT-TPA** were calculated at the B3LYP/6-31G** level in CH_2_Cl_2_. Table S1 gathers the charges accumulated by the constituting fragments (central core and DPA and TPA units) as oxidation occurs. For **ADT-DPA**^**•+**^, the charge for the radical cation species is withdrawn from the core (0.46e) and the DPA units (0.27e from each unit). For **ADT-TPA**^**•+**^, a slightly smaller charge is removed from the core (0.36e), whereas 0.32e are extracted from each TPA unit. Upon further oxidation to the dication, the charge extracted resides on both the core (1.14e) and the DPA units (0.43e each) for **ADT-DPA**^**2+**^, whereas it is mainly concentrated on the pendant TPA moieties (0.98e each) for **ADT-TPA**^**2+**^ (Table S1). This indicates that oxidation mainly involves the TPA units in **ADT-TPA**, whereas it implies both the ADT core and the peripheral DPA units for **ADT-DPA**. It should be stressed that, for both **ADT-DPA** and **ADT-TPA**, the ionization energies (IEs) required for going from the neutral molecule to the cation (IE1) and from the cation to the dication (IE2) are predicted to have relatively close values. The IE1 and IE2 values for **ADT-DPA** (**ADT-TPA**) were computed to be 4.50 (4.58) and 5.14 (4.96) eV, respectively. The larger separation between the IE1 and IE2 values for **ADT-DPA** (0.64 eV) compared to **ADT-TPA** (0.38 eV) may account for the structured two-electron first oxidation wave observed for **ADT-DPA** that coalesces into a non-structured broad oxidation wave for **ADT-TPA** (Figure 1a). The second oxidation wave observed for the later at 1.25 V corresponds to the oxidation of the ADT core. Furthermore, the higher IE1 value predicted for **ADT-TPA** is in agreement with the higher oxidation potential recorded for **ADT-TPA** compared to **ADT-DPA** (Table 1).

To evaluate the ability of **ADT-DPA** and **ADT-TPA** as HTMs, hole reorganization energies (λ) were computed at the B3LYP/6-31G** level in the gas phase (see the Supporting Information for full computational details). The λ values estimated for **ADT-DPA** and **ADT-TPA** are 0.460 and 0.241 eV, respectively. The λ values calculated for their constituting fragments are 0.193 eV for the ADT core and 0.309 and 0.274 eV for the DPA and TPA units, respectively. **ADT-TPA** therefore exhibits a λ value intermediate between those of the ADT core and the TPA units, whereas the λ value computed for **ADT-DPA** is higher than those of its constituting fragments and almost doubles that obtained for **ADT-TPA**. **ADT-TPA** actually holds a relatively low reorganization energy in the range found for other excellent p-type semiconducting organic materials,^37^ although higher than that obtained for the benchmark compound spiro-OMeTAD (0.139 eV).^28^ Therefore, **ADT-TPA** is considered a better candidate than **ADT-DPA** as the HTM for PSC devices owing to its significantly smaller λ value and appropriate energy level alignment with the valence band edge of the perovskite.

The novel HTMs show similar optical absorption patterns (Figure 1b and Table 1). Both ADT derivatives present a low-energy, intense (around 3 × 10^4^ M^–1^ cm^–1^) absorption band peaking at 413 and 431 nm for **ADT-DPA** and **ADT-TPA**, respectively, with a small shoulder at ca. 360 nm. The room-temperature emission spectra display a featureless broad band centered at 476 and 547 nm for **ADT-DPA** and **ADT-TPA**, respectively. Noticeably, **ADT-TPA** shows a large shift between the absorption and emission maxima of 130 nm.

A theoretical simulation of the absorption spectra of **ADT-DPA** and **ADT-TPA** (Figure S5) was performed based on the vertical energies and oscillator strengths calculated for the lowest-energy 80 singlet-excited electronic states (S_*n*_) at the B3LYP/6-31G** time-dependent DFT level in CH_2_Cl_2_. The theoretical spectrum for **ADT-DPA** notably reproduces the shape of the experimental spectrum with two intense bands centered at 468 and 280 nm and a shoulder between both bands (362 nm). A similar pattern with two intense absorption bands at 481 and 310 nm and a weaker, less-visible shoulder (ca. 350 nm) is predicted for **ADT-TPA**. Theoretical calculations therefore reproduce the red shift experimentally observed in passing from **ADT-DPA** to **ADT-TPA** (Figure 1b). For both molecules, the lowest-energy band is due to the S_0_ → S_1_ electronic transition mainly resulting from the HOMO → LUMO monoexcitation (see Figure S5 and Table S2). The shoulder is associated with the S_0_ → S_4_ electronic transition for **ADT-DPA**, and to the S_0_ → S_3_ and S_0_ → S_7_ electronic transitions for **ADT-TPA**. These transitions are of a π → π* nature and largely involve excitations centered on the core ADT moiety (Table S2 and Figure S6).

### Solar Cell Fabrication and Characterization

**Figure 3:**
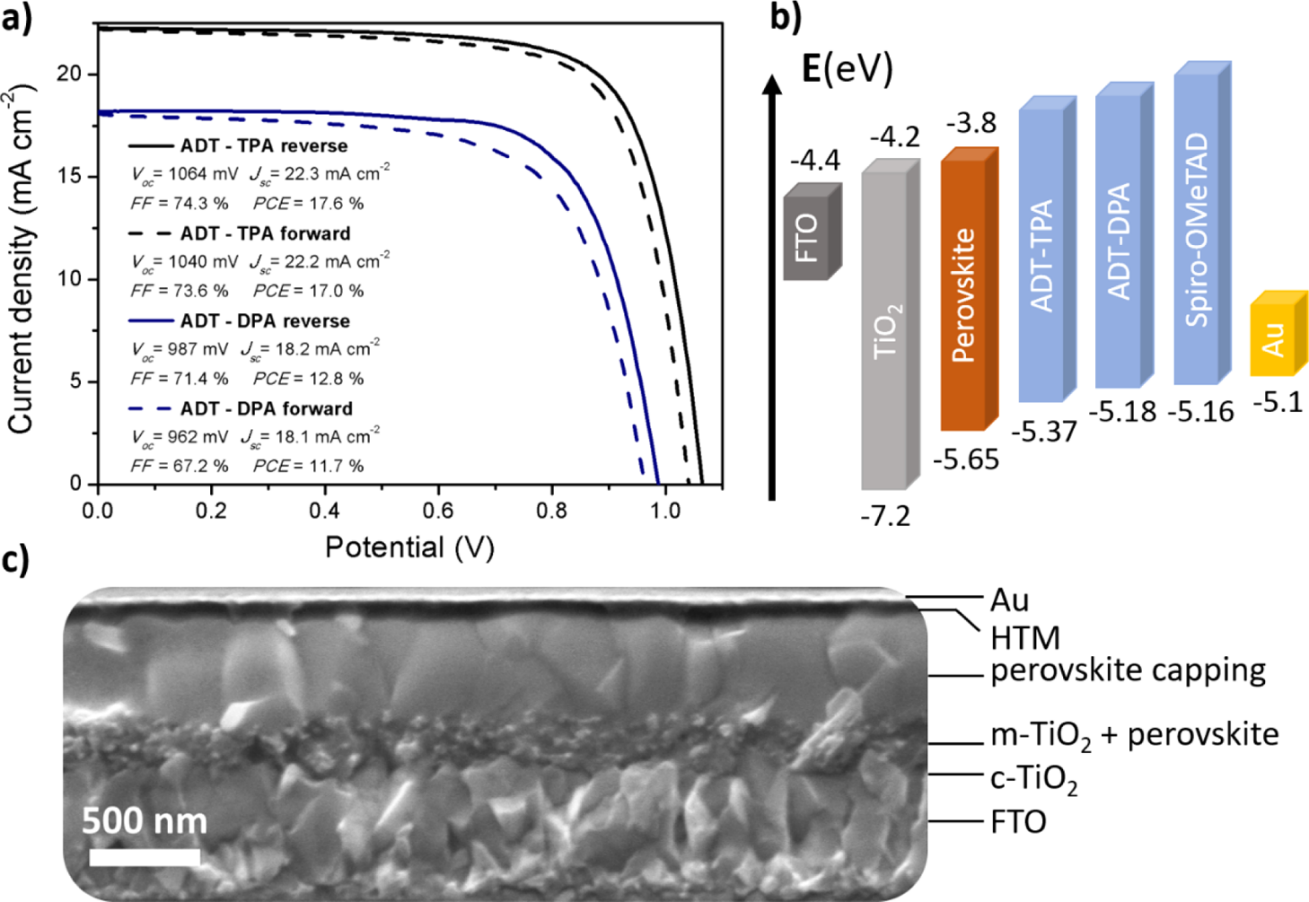
(a) Current density–voltage (*J*/*V*) curves for the champion devices recorded under 1 sun (100 mW cm^–2^) simulated sunlight. (b) Energy level diagram of different solar cell components. (c) Cross-sectional image of a PSC fabricated with **ADT-TPA** as the HTM.

The new compounds **ADT-DPA** and **ADT-TPA** were investigated as hole-extracting layers in PSCs and compared with the benchmark molecule spiro-OMeTAD. Devices with a conventional n–i–p configuration were fabricated from a stack of FTO/c-TiO_2_/m-TiO_2_/perovskite/HTM/Au (experimental details can be found in the Supporting Information). A mixed-ion perovskite with the (FAPbI_3_)_0.85_(MAPbBr_3_)_0.15_ composition was used as the light-absorbing material. The new HTMs as well as spiro-OMeTAD were chemically doped using *tert*-butylpyridine (Tbp), lithium bis(trifluoromethanesulfonyl)imide, and tris(2-(1*H*-pyrazol-1-yl)-4-*tert*-butylpyridine)cobalt(III)tri[bis(trifluoromethane)sulfonimide (FK 209 Co(III)-TFSI) as additives to increase their carrier mobility. The efficiencies of the fabricated devices were measured under 1 sun (100 mW cm^–2^) simulated sunlight. The current density/voltage (*J*/*V*) curves of the champion devices are shown in Figure 3a. The energy levels of the individual solar cell components are displayed in Figure 3b, and a SEM cross-sectional image of a finished device with **ADT-TPA** as the HTM is shown in Figure 3c. The thicknesses of the HTM layers for **ADT-TPA** and **ADT-DPA** were estimated to be around 90 and 40 nm, respectively.

**Figure 4:**
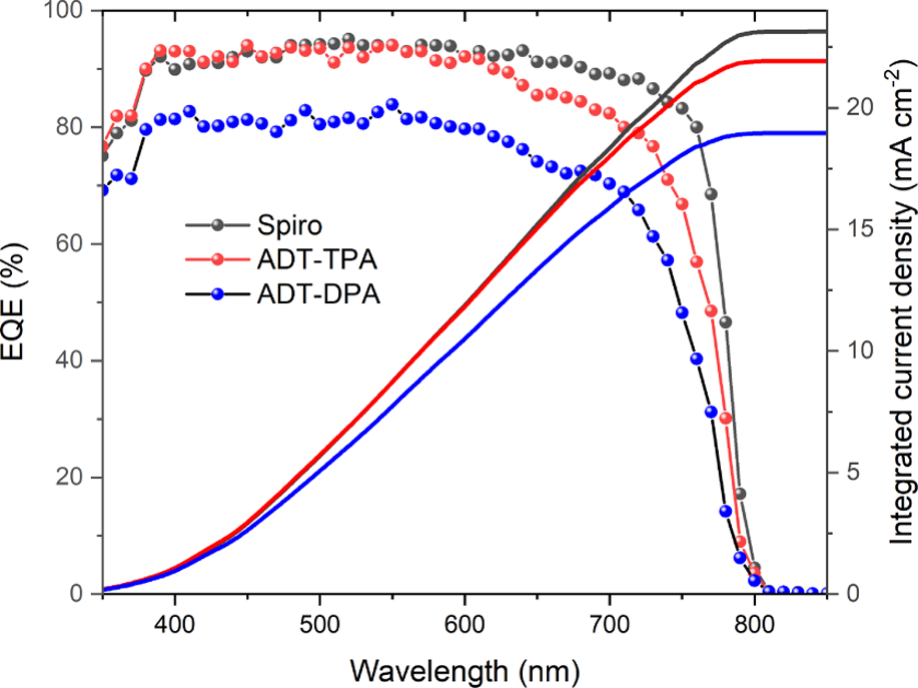
EQE spectrum and integrated current density of spiro-OMeTAD-, **ADT-TPA**-, and **ADT-DPA**-based PSCs.

Devices with **ADT-TPA** as the HTM were shown to perform significantly better than those with **ADT-DPA**. Reverse scans of **ADT-TPA** yielded efficiencies up to 17.6%, close to what was observed with the spiro-OMeTAD reference cell (Figure S8). To quantify the hysteresis between reverse and forward scans, the hysteresis index (HI) = (PCE_reverse_ – PCE_forward_)/PCE_reverse_ was calculated.^44^ For **ADT-TPA**, contributions from hysteresis (HI = 3.5%) were found to be small, much better than those observed for the reference spiro-OMeTAD (HI = 6.1%). The short circuit current (*J*_SC_) larger than 20 mA cm^–2^ was further confirmed by external quantum efficiency (EQE) measurements, as shown in Figure 4. Devices with **ADT-DPA**, however, show a significantly reduced open-circuit voltage (*V*_OC_) as well as a lower short-circuit current. Best efficiencies with **ADT-DPA** are therefore only 12.8%, measured in the reverse scan direction, showing a more pronounced hysteresis (HI = 8.6%) behavior. The underperformance of **ADT-DPA** devices with respect to **ADT-TPA** ones was initially attributed to poor layer formation: a cross-sectional image (Figure S9) unveils the presence of pin-holes that allow direct contact of the active layer with the gold electrode. These defects are suspected to be caused by the low solubility of **ADT-DPA** in chlorobenzene, the solvent employed for its deposition onto the perovskite layer. However, steady-state photoluminescence (PL) shows that both HTMs are able to effectively quench the PL of the perovskite (see Figure S10), which is indicative of interfacial hole transfer. Additionally, the process was monitored by time-resolved PL. The dynamics associated with the PL decay are consistent with a biexponential fit, where both spiro-OMeTAD and **ADT-TPA** show similar time constants, whereas **ADT-DPA** follows a much steeper decay (Figure S11). These results show that the new molecules can effectively quench the PL, thus representing efficient hole-transfer materials, similar in function to spiro-OMeTAD. Furthermore, the interfacial dynamics confirm the favorable hole transfer as it should be expected from the relative energy position of the HOMO level of the HTMs. One of the main critical issues of PSCs is that related to their stability in operational conditions.^45^

The study of our ADT-based devices under maximum-power-point-tracking allowed to monitor their stability at 1 sun illumination for up to 40 h compared to devices based on spiro-OMeTAD (Figure S12). **ADT-DPA** shows a dramatic efficiency drop after the first 10 h of operation to thereafter sustain an operation stability over 30% of the original values after 40 h. In comparison, devices based on spiro-OMeTAD maintain 90–100% of the original values of efficiency after 40 h, while those based on **ADT-TPA** show a loss in efficiency from 80 to 50% in 40 h. Despite the initial decrease, a gradual recuperation of the performance was observed, which might be correlated with the TPA content. Thermal stability of the solar cells was explored keeping the devices at 85 °C with a relative humidity of 20% in air conditions for up to 120 min (Figure S13). Initially, a performance decrease associated with the inter-penetration of spiro-OMeTAD and gold electrodes was observed; then, after 120 min, the performance of the PSCs dropped to 39% (Spiro), 30% (TPA), and 18% (DPA) of the initial efficiency, respectively.

**Figure 5:**
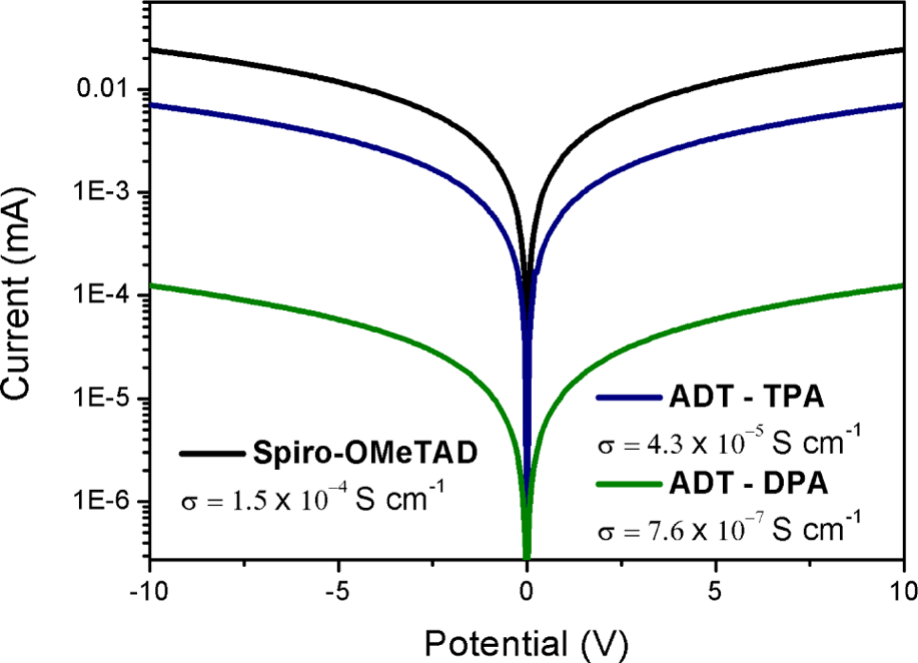
Semilogarithmic current/voltage plots of spiro-OMeTAD and ADT derivatives spin-coated onto substrates having interdigitating gold electrodes with a channel length of 2.5 μm. The calculated conductivity values are shown.

To compare the electrical properties of the different HTMs, conductivity measurements on substrates having interdigitating gold electrodes were performed. The HTMs were deposited using the same conditions as for the devices using 6 mol % FK209 as the dopant. A semilogarithmic representation of the current/voltage (*I*/*V*) curves and the corresponding conductivity values are shown in Figure 5. Spiro-OMeTAD was shown to have the largest conductivity (σ = 1.5 × 10^–4^ S cm^–1^) closely followed by **ADT-TPA** (σ = 4.3 × 10^–5^ S cm^–1^). Coherent with its poor performance in devices, HTM **ADT-DPA** showed conductivity (σ = 7.6 × 10^–7^ S cm^–1^) that is 2 orders of magnitude lower than that of **ADT-TPA**. These results are consistent with the reorganization energies calculated theoretically, from which **ADT-DPA** and **ADT-TPA** show λ values (0.460 and 0.241 eV, respectively) that are roughly 3.5 and 2 times higher than that of spiro-OMeTAD (0.139 eV). Moreover, the defect concentration of the hole-transporting layer was estimated from capacitance *versus* frequency measurements and density of states (DoS) calculations, plotting the DoS *versus* voltage (see Figure S14) that correlates with the trap-density distribution *versus* energy. From these data, the density of trap states for **ADT-DPA** and **ADT-TPA** in the band gap is similar but higher than that of spiro-OMeTAD. The band edge of **ADT-DPA** at HOMO is quenched compared to those of **ADT-TPA** and spiro-OMeTAD. This indicates that the carrier density of **ADT-DPA** in HOMO seems lower than those of **ADT-TPA** and spiro-OMeTAD, which is consistent with the conductivity data, and it could be the reason for the low photovoltaic properties of **ADT-DPA**. Altogether, the underperformance of **ADT-DPA** may be attributed to its low conductivity derived from a low carrier density in the HOMO, which in turn does not allow the efficient extraction of the holes photogenerated in the active layer. In contrast, **ADT-TPA**, showing a more planar molecular structure and a lower reorganization energy, exhibits conductivity and device efficiency similar to those of benchmark HTM spiro-OMeTAD.

## Conclusions

In summary, a new family of HTMs employing an ADT planar core has been synthesized. Depending on the appending functional groups linked to this novel building block, high-performance PSCs may be fabricated, exhibiting efficiencies (17.6%) close to that of the benchmark compound spiro-OMeTAD (18.1%). The synthetic availability of the ADT building block from very cheap precursors in three reaction steps makes ADT an appealing platform for synthesizing high-efficiency HTMs. In view of the obtained results, it is inferred that triphenylamine (TPA) pending groups are favored over diphenylamine (DPA) groups in terms of quality of the formed films and their enhanced conductivity. Aside from the solubility issues **ADT-DPA** exhibits, DFT calculations show that **ADT-TPA** features a flatter geometry that may enhance its solid-state stacking and, thereby, its conductivity. The TPA groups furthermore lead to a significantly lower reorganization energy for **ADT-TPA** that also favors hole extraction and conductivity.

## Experimental Section

Related materials, solvents, instruments, and detailed experimental and computational procedures can be obtained from the Supporting Information.

## Supporting Information Available

Experimental section; synthetic details and characterization; thermal properties; computational details; device fabrication and characterization; and ^1^H NMR, ^13^C NMR, and HRMS spectra (PDF)
