# Supplementary material for: Hole-Transporting Materials for Perovskite Solar Cells Employing an Anthradithiophene Core
Source: ACS Appl Mater Interfaces. 2021 Jun 9;13(24):28214–21. doi: 10.1021/acsami.1c05890 (PMC9205564; doi:10.1021/acsami.1c05890)
Supplement: Supplementary file 1 — am1c05890_si_001.pdf [file am1c05890_si_001.pdf]

Supporting Information

Hole-Transporting Materials for Perovskite Solar Cells Employing an Anthradithiophene Core

José Santos,*[a] Joaquín Calbo,[b] Rafael Sandoval-Torrientes,[a] Inés García-Benito,[a] Hiroyuki Kanda,[c] Iwan Zimmermann,[c] Juan Aragó,[b] Mohammad Khaja Nazeeruddin,*[c] Enrique Ortí,*[b] Nazario Martín,*[a,d]

[a] Facultad de Ciencias Químicas, Universidad Complutense de Madrid, Madrid, 28040, Spain.

[b] Instituto de Ciencia Molecular, Universidad de Valencia, Paterna, 46980, Spain.

[c] EPFL VALAIS, Sion, 1951, Switzerland.

[d] IMDEA-Nanociencia, Ciudad Universitaria de Cantoblanco, Madrid, 28049, Spain.

* Nazario Martín: E-mail: nazmar@ucm.es

* José Santos: E-mail: jose.santos@imdea.org

* Enrique Ortí: E-mail: enrique.orti@uv.es

* Mohammad Khaja Nazeeruddin: E-mail: mdkhaja.nazeeruddin@epfl.ch

Index

1. **Experimental Section** S2
2. **Synthetic Details and Characterization** S3
3. **Thermal Properties** S5
4. **Computational Details** S6
5. **Device Fabrication and Characterization** S12
6. **^{1}H NMR, ^{13}C NMR and HRMS spectra** S16
7. **References** S20

1. Experimental Section

General Methods. Chemicals and reagents were purchased from commercial suppliers and used as received. All solvents were dried according to standard procedures. Air-sensitive reactions were carried out under nitrogen atmosphere. The device preparation was done in a glovebox under nitrogen atmosphere. Flash chromatography was performed using silica gel (Fluorochem, Silicagel 60A, 40–63 micron). Analytical thin-layer chromatography (TLC) was performed using aluminum-coated Merck Kieselgel 60 F254 plates. NMR spectra were recorded on a Bruker Advance 300 (^{1}H: 400 MHz; ^{13}C: 101 MHz) spectrometer at 298 K using partially deuterated solvents as internal standards. Coupling constants (*J*) are denoted in Hz and chemical shifts (δ) in ppm. Multiplicities are denoted as follows: s = singlet, d = doublet, t = triplet, m = multiplet. UV-vis spectra were recorded in a Varian Cary 50 spectrophotometer. FT-IR spectra were recorded on a Bruker Tensor 27 (ATR device) spectrometer. Photoluminescence (PL) spectra were performed on a Fluorolog-3 HORIBA spectrofluorometer. Mass spectra matrix-assisted laser desorption ionization (coupled to a time-of-flight analyzer) experiments (MALDI-TOF) were recorded on a MAT 95 thermo spectrometer and a Bruker REFLEX spectrometer respectively. Thermogravimetric analysis (TGA) was performed using a TA Instruments TGAQ500 with a ramp of 10 °C min^{-1} under N_2 from 100 to 1000 °C. Differential scanning calorimetry (DSC) was run on a Discovery DSC from TA instruments. Three cycles were recorded under nitrogen, heating (until 400 °C) and cooling (50 °C) at 20 °C min^{-1} of scanning rate. Cyclic voltammetry (CV) experiments were performed in deaerated 0.1 M Bu_4NPF_6 dichloromethane (DCM) solutions at a scan rate of 100 mV s^{-1}. Glassy carbon was used as a working electrode and platinum wires as counter and reference electrodes. Before each measurement, solutions were deoxygenated with N_2. Ferrocene was added as an internal standard; its oxidation potential in DCM was set at 0.7 V vs. normal hydrogen electrode (NHE) and materials oxidation potentials were recalculated in reference to NHE. 1,5-Dichloroanthraquinone and mercaptoacetic acid were purchased from commercial suppliers. Compounds **4**[1] and **5**[2] were obtained according to previously reported procedures.

2. Synthetic Details and Characterization.

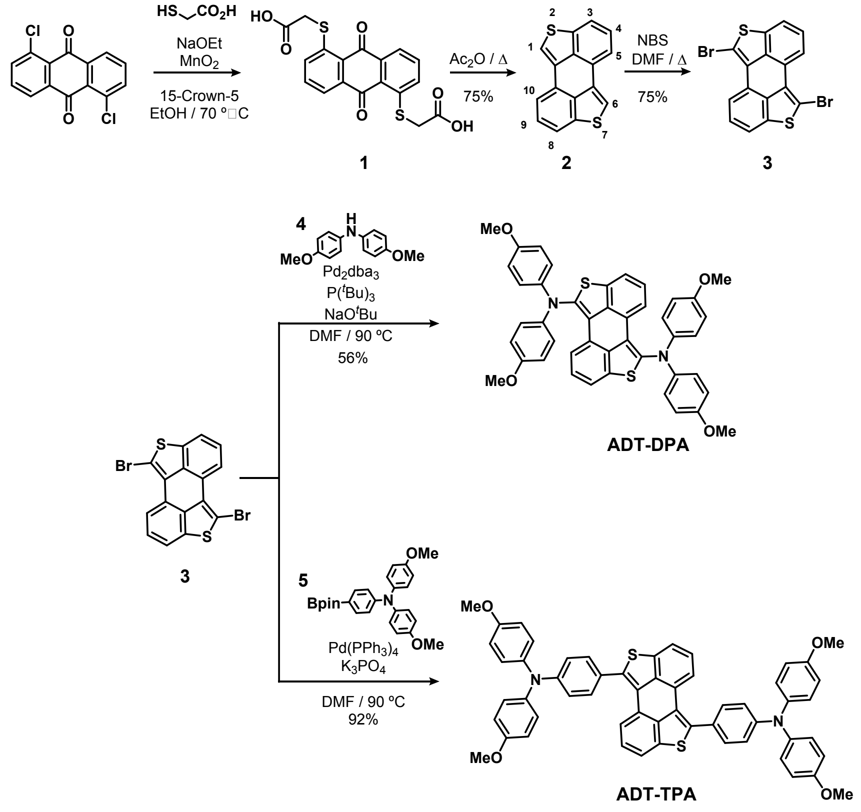

Scheme S1. Synthetic route for the preparation of the new HTMs based on ADT.

Anthra[1,9-*bc*:5,10-*b′c′*]dithiophene (2)

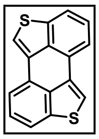

Into a 100 mL 2-neck round-bottom flask fitted with a condenser, 1,5-dichloroanthraquinone (5.00 g, 18.0 mmol) was dissolved in a sodium ethoxide ethanol solution (43 mL, 21% w/w). Thereafter, mercaptoacetic acid (3.64 g, 39.6 mmol) was added in portions at 22 °C, followed by the addition of MnO_2 (0.85 g, 9.85 mmol) and 15-crown-5-ether (85.0 mg, 0.40 mmol). The resulting mixture was stirred at 70 °C for 14 h. Upon cooling to room temperature, the mixture was gradually poured into H_2O (400 mL), stirred mixture was then acidified to pH < 3 with 2 M hydrochloric acid. The formed precipitate was collected by filtration, washed with water, and dried under vacuo to give compound **1** as black solid. The black solid was used in the next step without further purification. Compound **1** (6.89 g, 17.5 mmol) was

suspended in acetic anhydride (120 mL) and was refluxed for 3 h. Without cooling, the precipitate was collected by filtration and then redissolved in hot *n*-pentane (250 mL). After removing the solvent under reduced pressure, the reaction crude was purified by flash chromatography (silica gel using hexane/DCM 4:1 as eluent) yielding **2** as a yellow solid (3.62 g, 75%). ^{1}H NMR (400 MHz, $CDCl_3$) δ: 7.70 (dd, *J* = 2.9, 0.7 Hz, 2H), 7.68 (dd, *J* = 3.6, 0.7 Hz, 2H), 7.63 (s, 2H), 7.40–7.35 (m, 2H) ppm; ^{13}C NMR (101 MHz, $CDCl_3$) δ: 140.0, 137.5, 132.1, 128.6, 126.1, 121.4, 118.0, 117.3 ppm; MS (MALDI-TOF) m/z: 264.0.

1,6-Dibromoanthra[1,9-*bc*:5,10-*b'c'*]dithiophene (3)

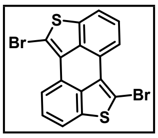

Into a solution of **2** (0.28 g, 1.08 mmol) in dry DMF (35 mL), a solution of *N*-bromosuccinimide (0.67 g, 3.77 mmol) in DMF (6 mL) was dropwise added. The resulting mixture was stirred at 110 ºC until totally dissolved. Thereafter, the mixture was cooled down to 80 ºC during 1h. The formed precipitate was collected by filtration, washed with acetone, and dried under vacuum to give rise to **3** as a pale yellow solid (0.32 g, 75%). ^{1}H NMR (400 MHz, DMSO-d^6) δ: 8.66 (d, *J* = 7.7 Hz, 2H), 7.97 (d, *J* = 7.9 Hz, 2H), 7.59 (t, *J* = 8.0 Hz, 2H) ppm; ^{13}C NMR could not be recorded due to compound's lack of solubility; HRMS (MALDI-TOF): m/z calculated for $C_{16}H_6Br_2S_2$: 419.8, found: 419.9.

ADT-DPA

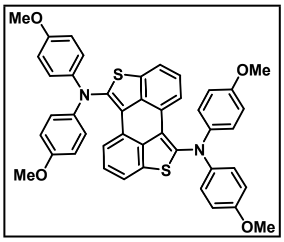

Into a 100 mL 2-neck round-bottom flask fitted with a condenser, **3** (120 mg, 0.30 mmol), bis(4-methoxyphenyl)amine (150 mg, 0.67 mmol), and Pd_2dba_3 (13.0 mg, 0.01 mmol) were dissolved in dry DMF (25 mL) under nitrogen atmosphere. The mixture was degassed for 30 min. *tert*-BuONa (84.0 mg, 0.88 mmol) was then added in one portion and the resulting mixture stirred at 110 °C for 5 h. Upon cooling to room temperature, the solvent was removed under reduced pressure and the reaction crude purified by flash chromatography (silica gel, DCM) to afford **ADT-DPA** as a yellow solid (120 mg, 56%). ^{1}H NMR (400 MHz, THF-d^8) δ: 7.82 (d, *J* = 7.8 Hz, 2H), 7.44 (d, *J* = 8.0 Hz, 2H), 7.14–7.06 (m, 10H), 6.86–6.77 (m, 8H), 3.72 (s, 12H) ppm; ^{13}C NMR (101 MHz, THF-d^8) δ: 157.3, 144.8, 141.4, 137.8, 137.6, 128.5, 126.7, 124.3, 124.0, 121.5, 121.1, 115.5, 55.7 ppm; FTIR (neat): 3849, 3726, 3680, 3650, 3620, 2950, 2836, 1741, 1645 1546, 1504, 1462, 1406, 1358, 1242, 1179, 1035, 825, 778 cm^{-1}; HRMS (MALDI-TOF): m/z calculated for $C_{44}H_{34}N_2O_4S_2$: 718.1955, found: 718.1923.

ADT-TPA

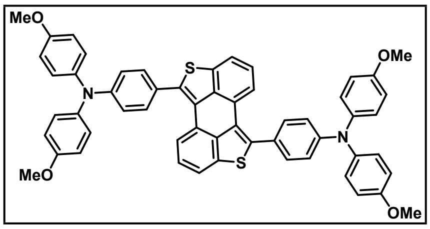

Into a 100 mL 2-neck round-bottom flask fitted with a condenser, **3** (200 mg, 0.47 mmol), 4-(4,4,5,5-tetramethyl-1,3,2-dioxaborolan-2-yl)-N,N-bis(4-methoxyphenyl)aniline (510 mg, 1.20 mmol), and $Pd(PPh_3)_4$ (54.0 mg, 0.05 mmol) were dissolved in dry DMF (25 mL) under nitrogen atmosphere. After degassing the mixture for 30 min, K_3PO_4 (1.20 g, 5.68 mmol) was added in one portion and the reaction stirred at 100 °C for 2 h. Upon cooling to room temperature, the reaction was quenched with H_2O (20 mL). The formed precipitate was filtered off and sequentially washed with H_2O and MeOH. Thereafter, the precipitate was redissolved in DCM (50 mL), the solvent removed under reduced pressure and the reaction crude purified by flash chromatography (silica gel, DCM) to give a pale orange solid (380 mg, 92%). ^{1}H NMR (400 MHz, THF-d^8) δ: 7.58–7.53 (m, 4H), 7.43–7.39 (m, 4H), 7.18–7.14 (m, 8H), 7.12–7.06 (m, 2H), 7.01–6.96 (m, 4H), 6.93–6.88 (m, 8H), 3.77 (s, 12H) ppm; ^{13}C NMR (101 MHz, THF-d^8) δ: 158.0, 150.8, 141.3, 140.1, 139.8, 138.4, 131.2, 130.4, 128.4, 127.2, 126.7, 126.1, 121.1, 120.1, 115.8, 55.8 ppm; FTIR (KBr): 3008, 2945, 2832, 1605, 1546, 1499, 1464, 1405 1319, 1288, 1234, 1173, 1100, 1028, 914, 879, 823, 767, 721, 677, 649, 601, 573 cm^{-1}; HRMS (MALDI-TOF): m/z calculated for $C_{56}H_{42}N_2O_4S_2$: 870.2581, found: 870.2584.

3. Thermal Properties

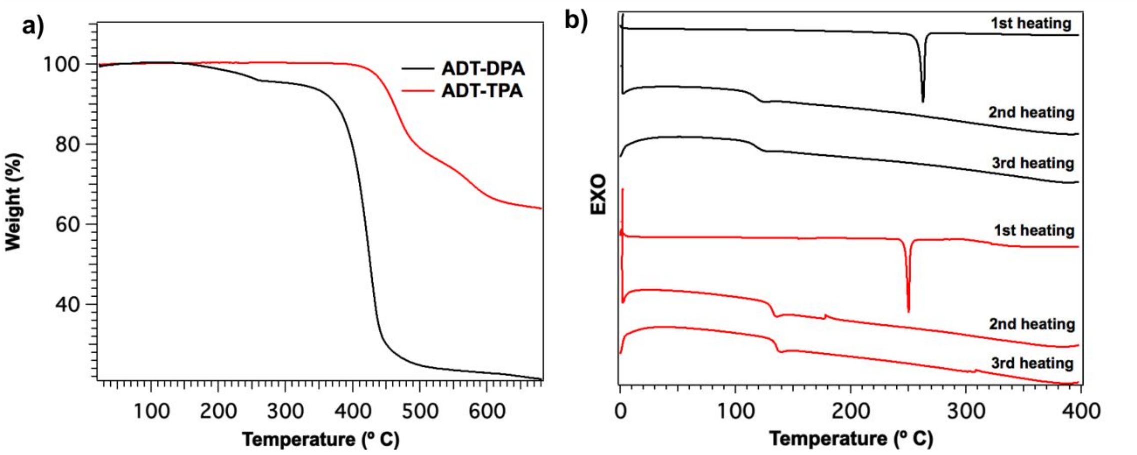

Figure S1. a) Thermogravimetric analysis of the HTMs under nitrogen at a heating rate of 10 °C min^{-1}. b) Differential scanning calorimetry of **ADT-TPA** (red) and **ADT-DPA** (black) under nitrogen at a heating rate of 20 °C min^{-1} (1st, 2nd, and 3rd cycles).

4. Computational Details

Quantum-chemical calculations were carried out with the Gaussian 16 (revision A.03) software package.[3] All the calculations were performed within the density functional theory (DFT) framework using the B3LYP functional[4] and the 6-31G** basis set.[5] Solvent effects were considered within the self-consistent reaction field (SCRF) theory by using the polarized continuum model (PCM) approach.[6] C_2-symmetry constraints were imposed during the optimization of the **ADT-DPA** and **ADT-TPA** HTMs, whereas C_{2h} constraints were used to calculate the isolated ADT core. The DPA and TPA substituent groups as well as the spiro-OMeTAD reference molecule were also optimized under C_2 symmetry. Vertical electronic transition energies to the lowest-energy singlet excited states of **ADT-DPA** and **ADT-TPA** were computed by using the time-dependent DFT (TDDFT) approach.[7] The lowest 80 singlet excited states were calculated at the B3LYP/6-31G** level using the ground-state optimized geometries. Geometry optimizations in gas phase of the radical cations of **ADT-DPA** and **ADT-TPA**, the ADT core, the DPA and TPA terminal groups, and spiro-OMeTAD were also performed to evaluate the reorganization energy (λ). The procedure used to evaluate λ is explained below. To investigate the oxidation processes, the ionization energies upon to the dication species were estimated in solution. Radical cations were treated as open-shell systems and computed within the spin-unrestricted DFT approximation at the UB3LYP/6-31G** level in the presence of CH_2Cl_2. Additionally, dication species were also computed in CH_2Cl_2. Molecular orbitals were plotted using the Chemcraft 1.6 software with isovalue contours of ±0.03 a.u.[8]

Molecular structures. Figure S2 displays the optimized structures of the two-limbed HTMs. Figure S3 shows the B3LYP/6-31G**-optimized values calculated for selected bond lengths of the ADT core and the **ADT-DPA** and **ADT-TPA** HTMs.

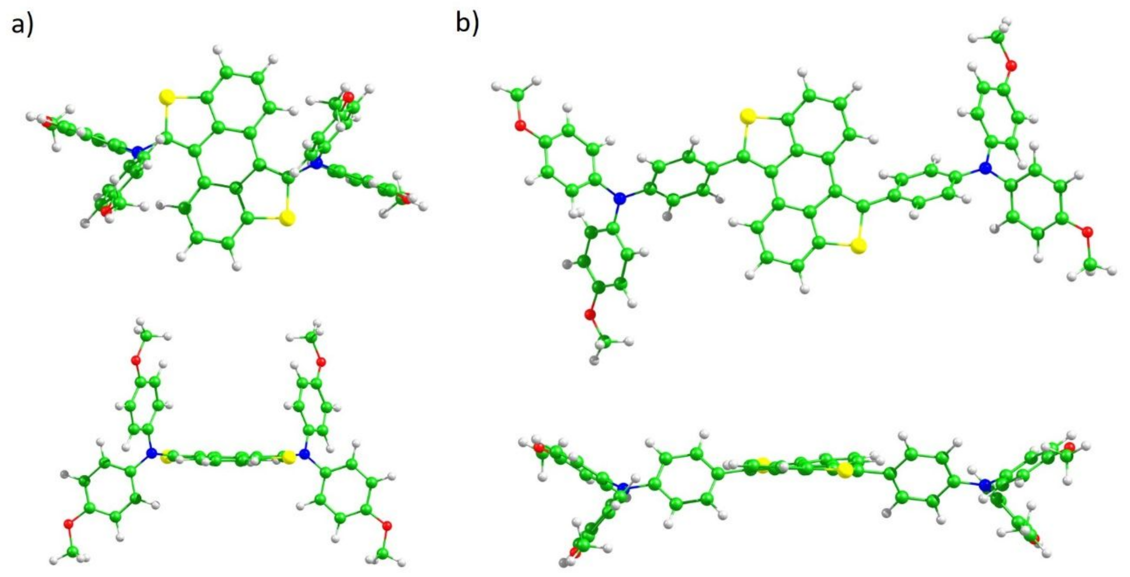

Figure S2. Top and side views of the B3LYP/6-31G**-optimized geometries calculated in CH_2Cl_2 solution for the **ADT-DPA** (a) and **ADT-TPA** (b) HTMs.

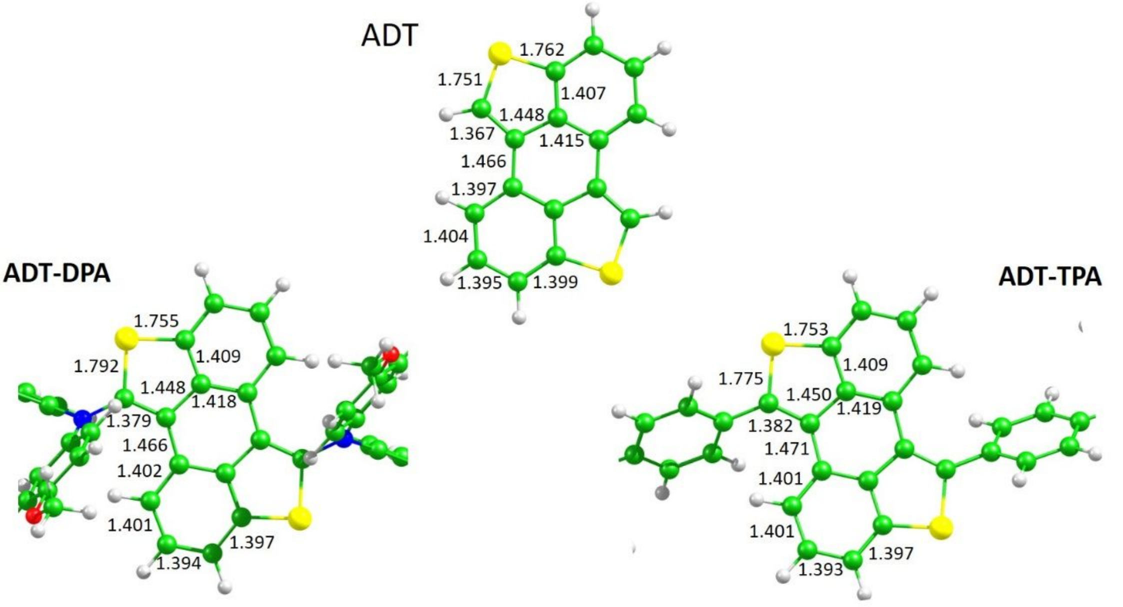

Figure S3. Optimized bond lengths (in Å) calculated at the B3LYP/6-31G** level, in CH_2Cl_2 solution, for the ADT core and for the **ADT-DPA** and **ADT-TPA** compounds. For the latter, the terminal DPA and TPA groups have been partially omitted and only the bond lengths calculated for the ADT core are quoted.

Oxidized species. B3LYP/6-31G** calculations in CH_2Cl_2 were used to investigate the molecular structure and charge distribution of the two-branched **ADT-DPA** and **ADT-TPA** HTMs in different oxidation states, monocation and dication. The radical cation species was computed as a doublet open-shell species. The dication was calculated both as a singlet closed-shell species, in which both electrons are extracted from the same orbital, and as a triplet open-shell species, in which the electrons are extracted from different orbitals. The singlet state was found to be more stable than the triplet state by 0.28 eV for **ADT-DPA** whereas the triplet is the most stable by 0.13 eV compared to the singlet for **ADT-TPA**. Table S1 gathers the Mulliken atomic charges computed for the central ADT cores and the peripheral DPA and TPA groups of the different oxidized species of **ADT-DPA** and **ADT-TPA** in their most stable electronic states.

Table S1. Mulliken atomic charges (in e) computed for the central ADT core and the DPA and TPA peripheral units of **ADT-DPA** and **ADT-TPA** in different oxidized states.

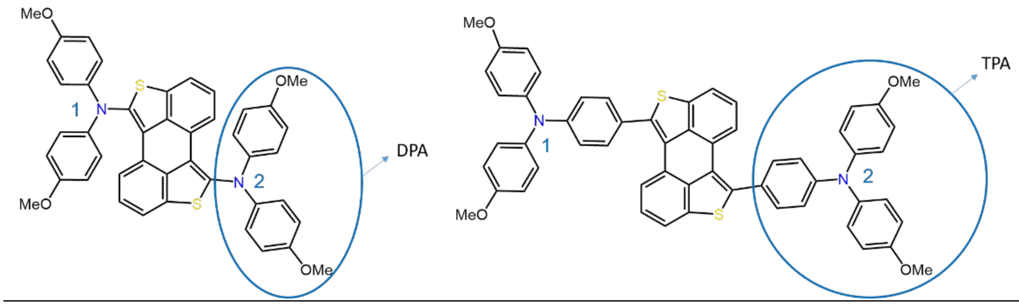

Molecular fragment	Neutral	Cation	Dication
**ADT-DPA**			
ADT	0.212	0.669	1.140
DPA-1	–0.106	0.165	0.430
DPA-2	–0.106	0.165	0.430
**ADT-TPA**			
ADT	–0.251	0.112	0.042
TPA-1	0.125	0.444	0.979
TPA-2	0.125	0.444	0.979

Figure S4 represents the potential energy surfaces for the neutral and cation states of two molecules (labelled as 1 and 2) involved in an intermolecular charge transfer process. The intramolecular reorganization energy (λ) consists of two terms related to the geometry relaxation energies of one molecule going from the fully relaxed ground state of the neutral species to the cation state (Figure S4, left) and a neighbouring molecule evolving in the opposite way (Figure S4, right),

$$\lambda = \lambda_1 + \lambda_2 \tag{1}$$

$$\lambda_1 = E\ (M1) - E\ (M1^+) \tag{2}$$

$$\lambda_2 = E\ (M2^+) - E\ (M2) \tag{3}$$

Here, E(M1) and E($M1^+$) for molecule 1 are the energies of the positively charged molecule (the cation) at the equilibrium geometry of the neutral molecule and the relaxed cation, respectively, and E($M2^+$) and E(M2) for molecule 2 are, accordingly, the energies of the neutral molecule at the equilibrium geometry of the cation and the neutral molecule, respectively.

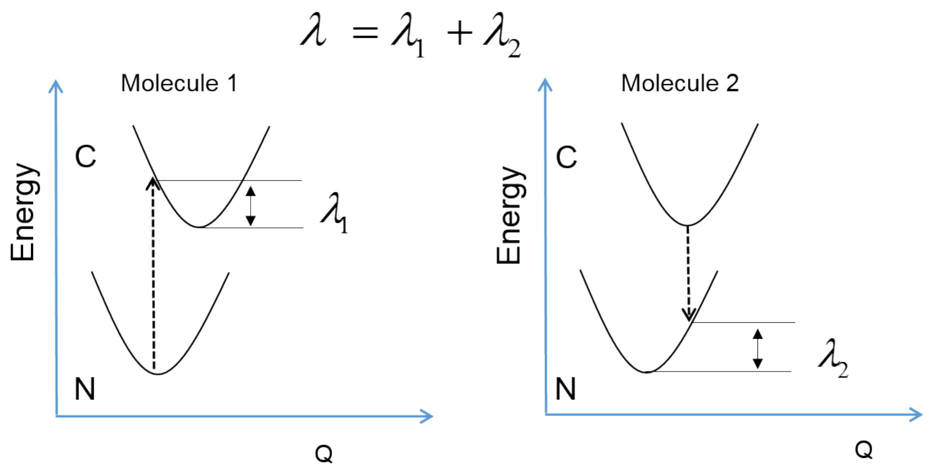

Figure S4. Scheme of the potential energy surfaces of the neutral state (N) and the cation state (C) for two molecules (1 and 2) involved in a charge (hole) transfer process. λ_1 and λ_2 are the two contributions to the total intramolecular reorganization energy (λ).

Electronic spectra. Figure S5 displays the theoretical simulation of the absorption spectra calculated for **ADT-DPA** and **ADT-TPA** from the electronic transitions computed at the TDDFT B3LYP/6-31G** level in CH_2Cl_2 solution. Table S2 gathers the vertical excitation energies (*E*), the oscillator strengths (*f*), and the electronic descriptions in terms of one-electron molecular orbital excitations calculated for the most relevant $S_0 \rightarrow S_n$ electronic transitions of **ADT-DPA** and **ADT-TPA** in CH_2Cl_2. Figure S6 depicts the topology and energy of the frontier molecular orbitals participating in the $S_0 \rightarrow S_n$ transitions quoted in Table S2.

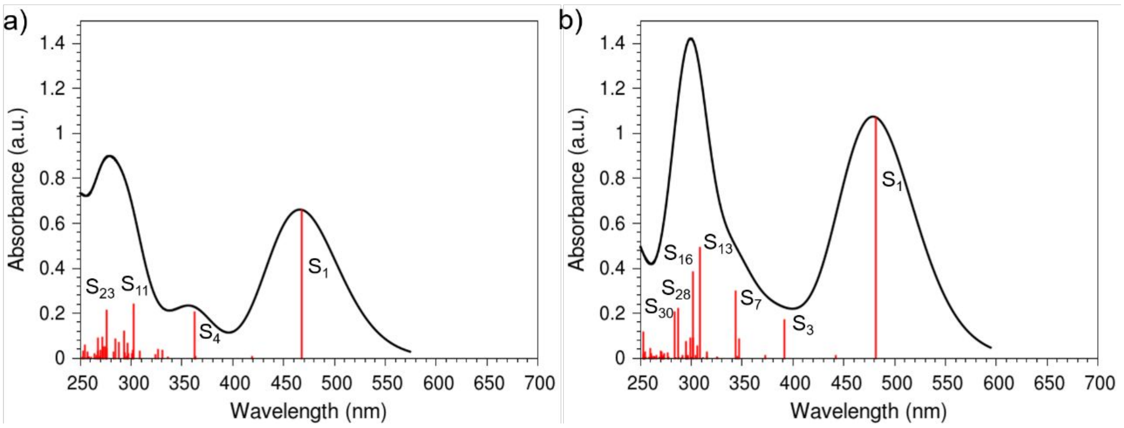

Figure S5. Stick and convoluted absorption spectra computed for **ADT-DPA** (a) and **ADT-TPA** (b) at the TDDFT B3LYP/6-31G** level in CH_2Cl_2. The TDDFT stick spectra were convoluted with Gaussian functions of full-width at half maximum (FWHM) of 0.20 eV.

Table S2. Lowest-energy singlet excited states calculated at the TDDFT B3LYP/6-31G** level for **ADT-DPA** and **ADT-TPA** in CH_2Cl_2 solution. Vertical excitation energies (*E*), oscillator strengths (*f*) and dominant monoexcitations with contributions (within parentheses) greater than 10%.

State	*E* (eV / nm)	*f*	Description[a]
**ADT-DPA**			
S_1	2.65 / 468	0.659	H → L (96)
S_4	3.42 / 362	0.205	H–2 → L (93)
**ADT-TPA**			
S_1	2.58 / 481	1.068	H → L (98)
S_3	3.17 / 392	0.168	H–2 → L (97)
S_7	3.61 / 343	0.299	H–1 → L+1 (59)<br>H–1 → L+3 (11)<br>H → L+2 (26)

[a] H and L denotes HOMO and LUMO, respectively.

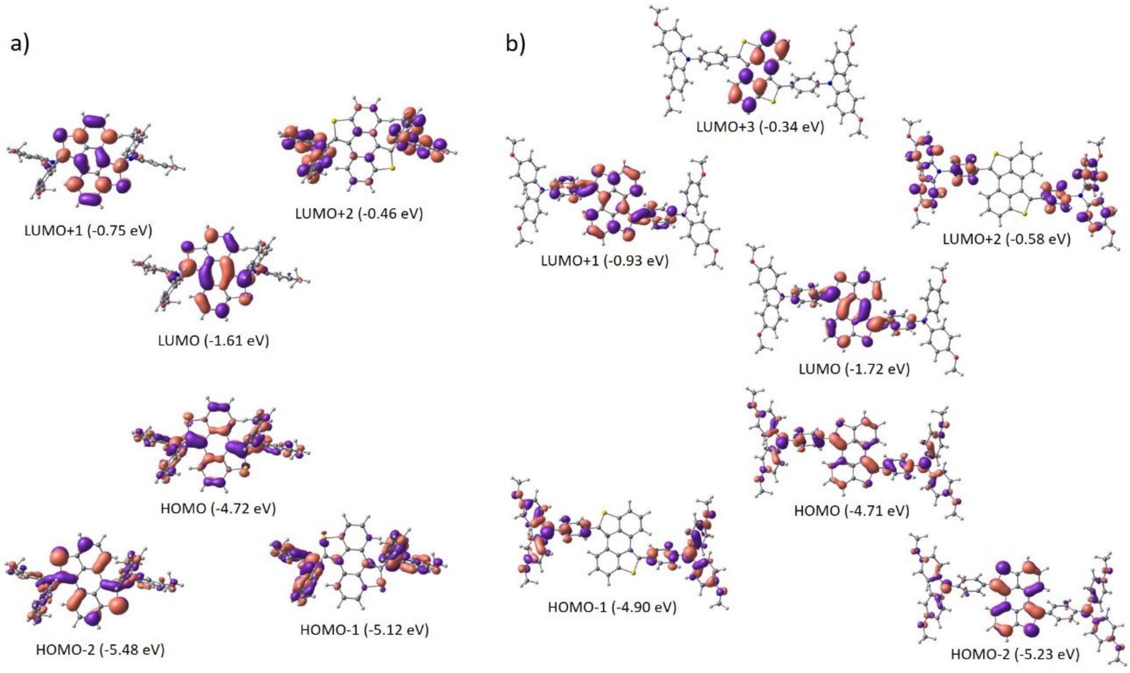

Figure S6. Isovalue contours (±0.03 a.u.) and energies calculated at the B3LYP/6-31G** level in CH_2Cl_2 for selected molecular orbitals of **ADT-DPA** (a) and **ADT-TPA** (b).

5. Device fabrication and characterization

5.1 Device preparation

Devices were fabricated onto fluorine-doped tin oxide (FTO) coated glass substrates (NSG-10). The substrates were cut and cleaned by sequential treatment with a 2% aqueous Helmanex solution and isopropanol in an ultrasonic bath for 20 and 10 minutes, respectively. The titania blocking layer (c-TiO_2) was applied by spray pyrolysis from a precursor solution of 0.2 mL titanium diisopropoxide bis(acetylacetonate) in 5 mL ethanol at 450 °C in ambient atmosphere. A 150 nm-thick mesoporous TiO_2 layer (m-TiO_2) was obtained by spin coating a titanium dioxide paste (30 nm particle size) from GreatCell Solar diluted in ethanol. The substrates were then sintered at 450 °C for 30 min. For the perovskite precursor solution 508 mg of PbI_2 (TCI), 68 mg PbI_2 (TCI), 180.5 mg formamidinium iodide (GreatCell Solar) and 20.7 mg methylammonium bromide (GreatCell Solar) were dissolved in a 1:4 mixture of DMSO:DMF. The perovskite solution was spun at 5000 rpm for 30 s using a ramp of 2000 rpm s^{-1}. 15 s prior to the end of the spin-coating sequence 100 µL of chlorobenzene were poured onto the spinning substrate. Afterwards, the substrates were transferred onto a hot plate and annealed at 100 °C for 50 min. The hole-transporting materials were dynamically spin-coated from solutions at 4000 rpm for 25 s. The hole transporters were dissolved in chlorobenzene with concentrations of 0.06 M for spiro-OMeTAD and 0.03 M for **ADT-DPA** and **ADT-TPA**. *Tert*-butylpyridine (Tbp), lithium bis(trifluoromethanesulfonyl)imide (Li-TFSI) and tris(2-(1*H*-pyrazol-1-yl)-4-*tert*-butylpyridine)cobalt(III) tri[bis(trifluoromethane)sulfonimide (FK 209 Co(III)-TFSI) were added as additives. Equimolar amounts of additives were added for all hole transporters: 330 mol% Tbp, 50 mol% Li-TFSI from a 1.8 M stock solution in acetonitrile and 6 mol% FK209 from a 0.25 M stock solution in acetonitrile. All HTM solutions were filtered using a syringe filter with a pore size of 0.22 µm prior to use. For the top electrodes 70 nm of gold was thermally evaporated under high vacuum.

5.2 Solar cell characterization

SEM images were recorded using a FEI Teneo microscope. Conductivity measurements were performed on substrates having interdigitating gold electrodes with a channel length of 2.5 µm. HTMs were spin-coated onto the substrates using the same conditions and concentrations as for the devices. 6 mol% FK209 was used as dopant. Current density-voltage characteristics were measured in air under AM 1.5 simulated sunlight (100 mW cm^{-2}) with a potentiostat (Keithley). The light intensity was measured for calibration with an NREL certified KG5 filtered Si reference diode. The solar cells were masked with a metal aperture of 0.16 cm^2 to define the active area. The current density-

voltage curves were recorded scanning at 10 mV s^{-1}. MPPT study was performed under 1 sun illumination with nitrogen atmosphere with RH0% at 25 °C.

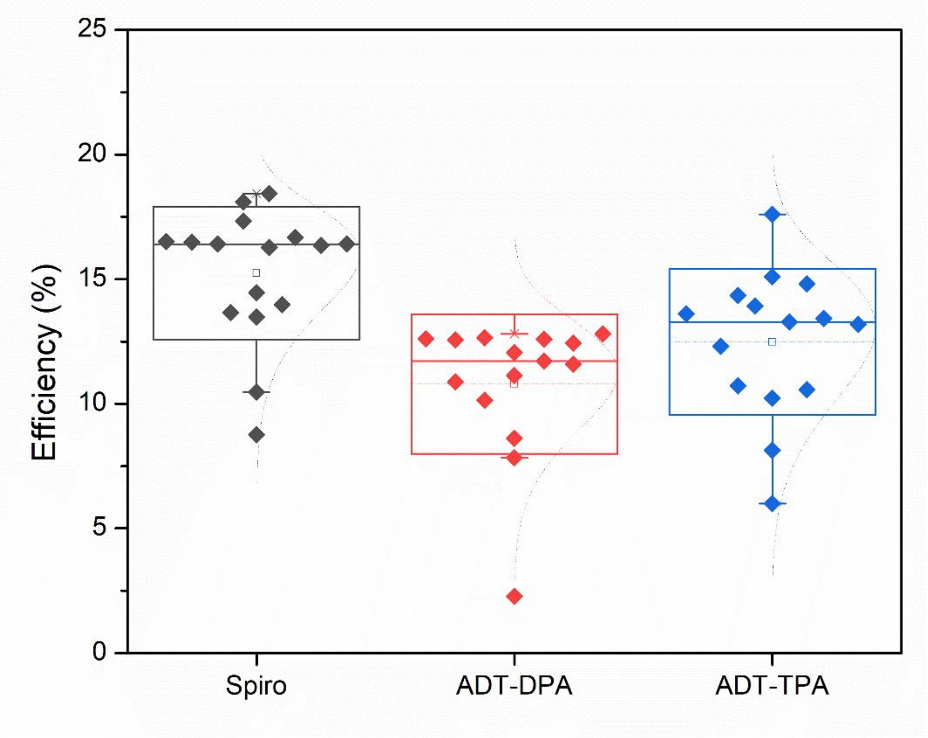

Figure S7. Performance statistics (averages, standard deviations) for Spiro-OMeTAD, **ADT-TPA** and **ADT-DPA**-based devices.

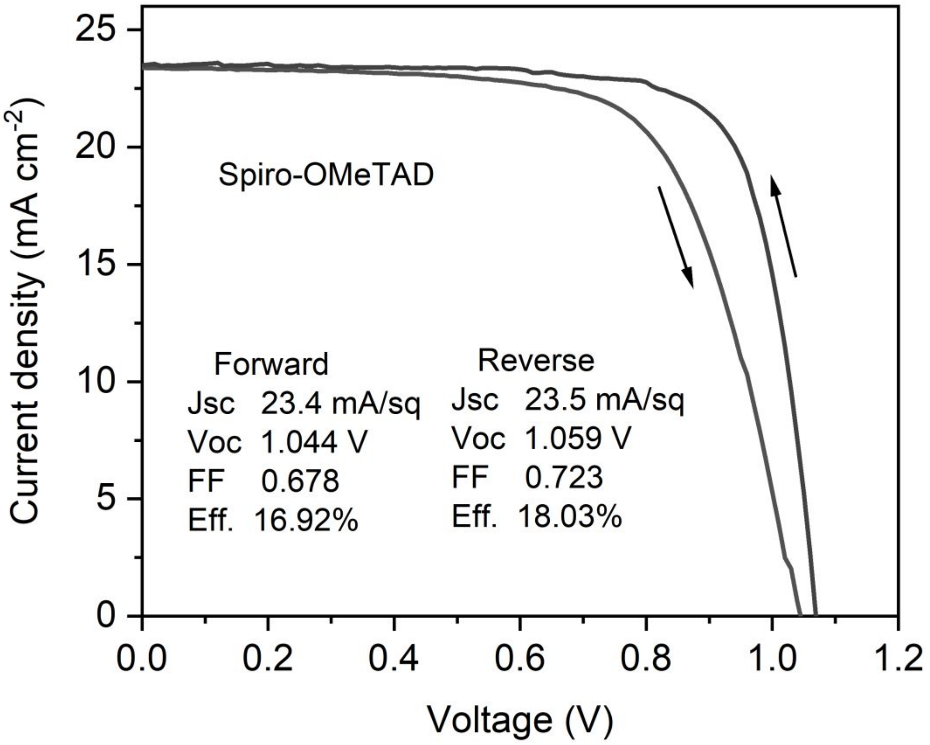

Figure S8. *J*/*V* curve of the reference cell fabricated with spiro-OMeTAD as HTM.

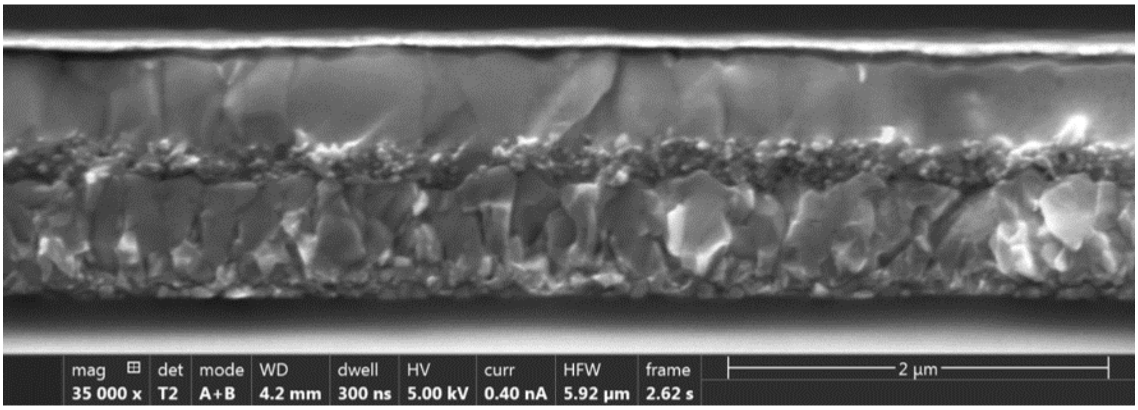

Figure S9. Cross-sectional image of a device fabricated with **ADT-DPA** as HTM.

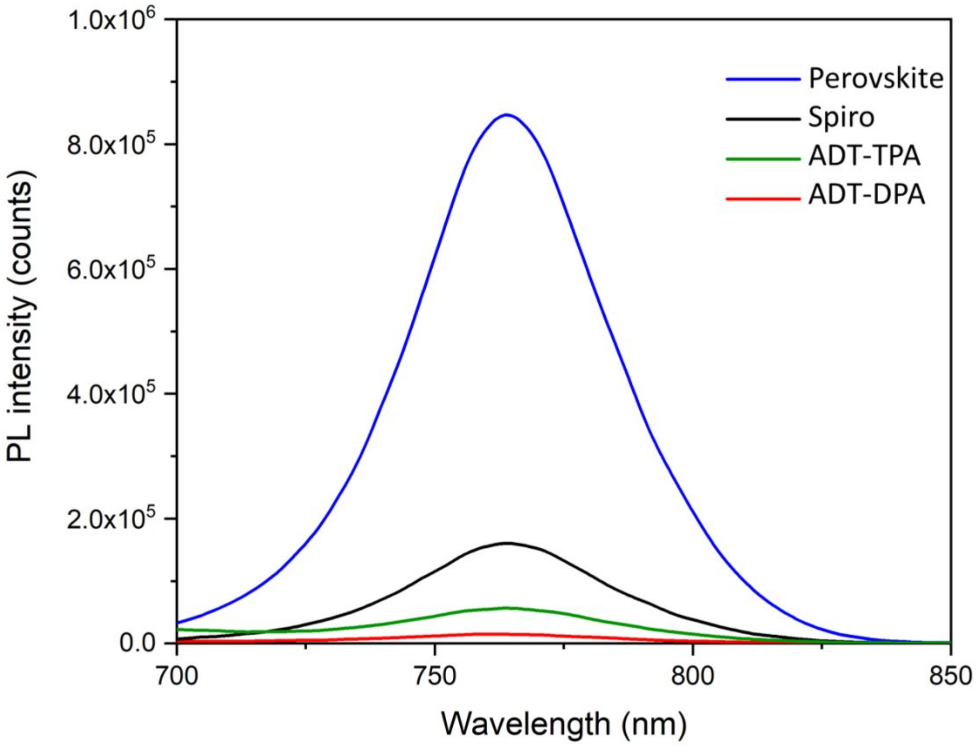

Figure S10. Photoluminescence spectra (excitation at 600 nm) of pristine perovskite (PVK1), and of spiro-OMeTAD, **ADT-TPA** and **ADT-DPA** interfaced with PVK1.

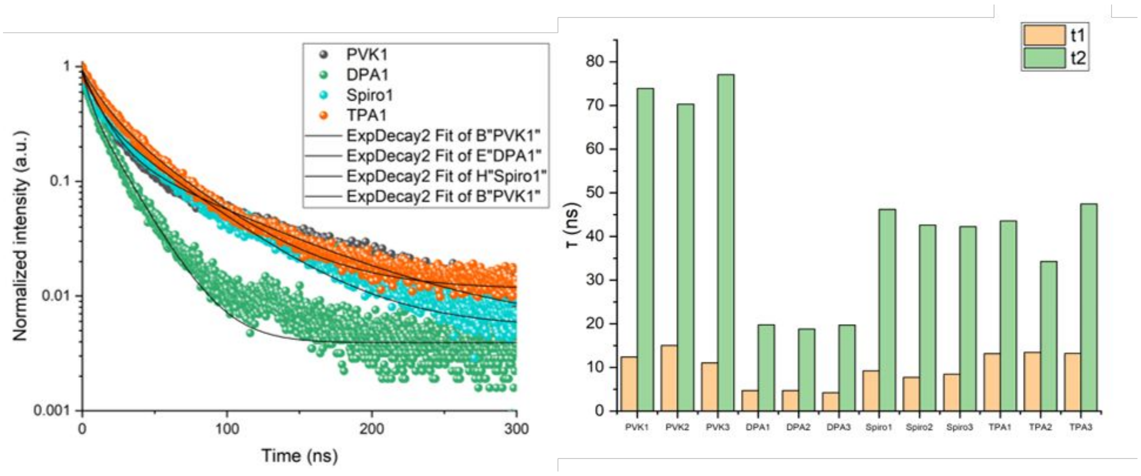

Figure S11. PL decay (left) and histograms (right) of t1 and t2 for pristine perovskite (PVK1), spiro-OMeTAD, **ADT-TPA** (TPA1) and **ADT-DPA** (DPA-1). A double exponential decay for the fitting was employed: y = y0 + A1*exp(-(x-x0)/t1) + A2*exp(-(x-x0)/t2).

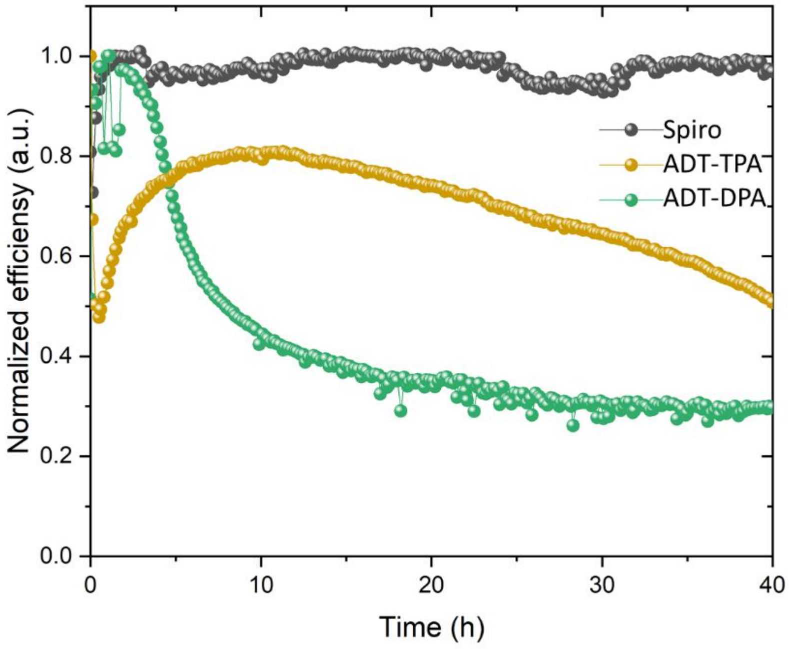

Figure S12. Normalized efficiency *vs.* time for perovskites solar cells with **ADT-TPA**, **ADT-DPA** and Spiro-OMeTAD under 1 sun illumination at 25 °C.

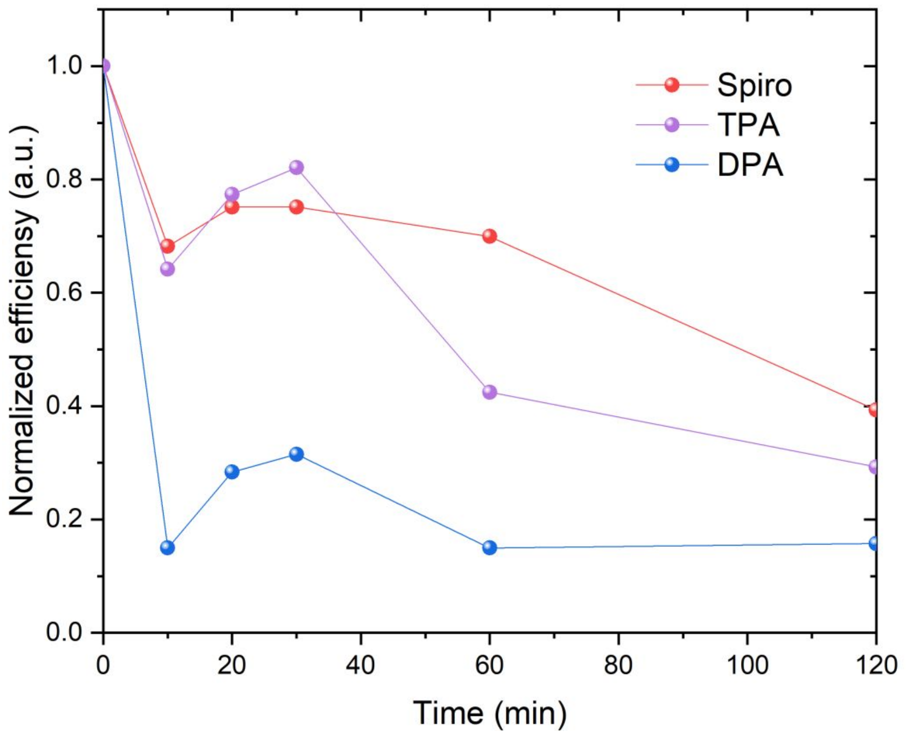

Figure S13. Normalized efficiency *vs.* time at 85 °C (RH20% in air) for perovskites solar cells with **ADT-TPA**, **ADT-DPA** and Spiro-OMeTAD.

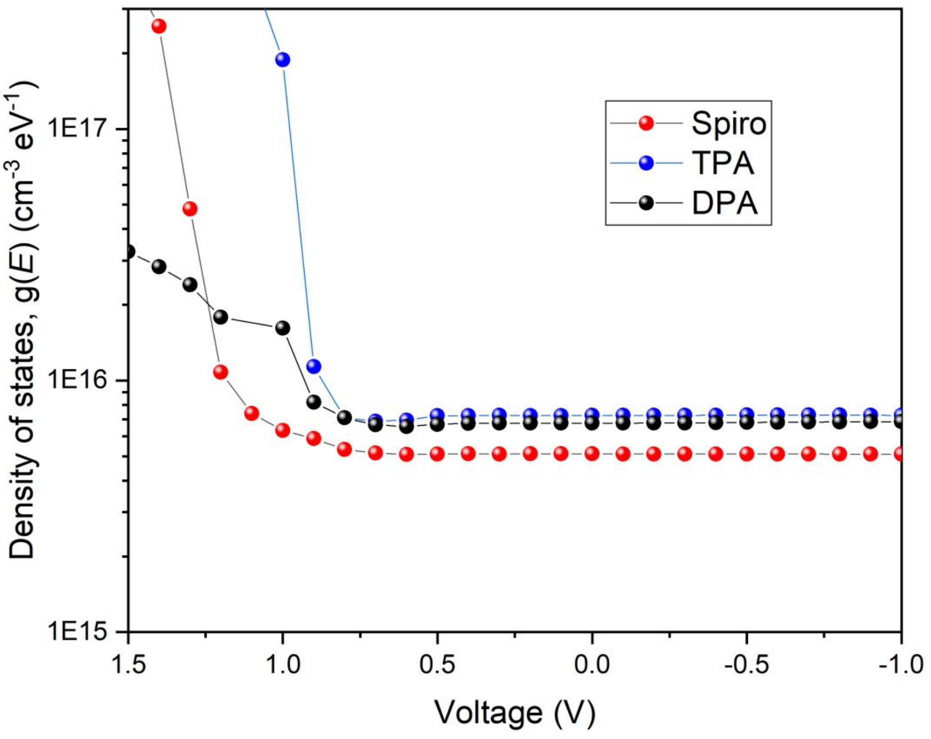

Figure S14. Density of states *vs.* voltage curves for ADT-TPA, **ADT-TPA** and spiro-OMeTAD.

6. ^{1}H NMR, ^{13}C NMR and HMRS spectra

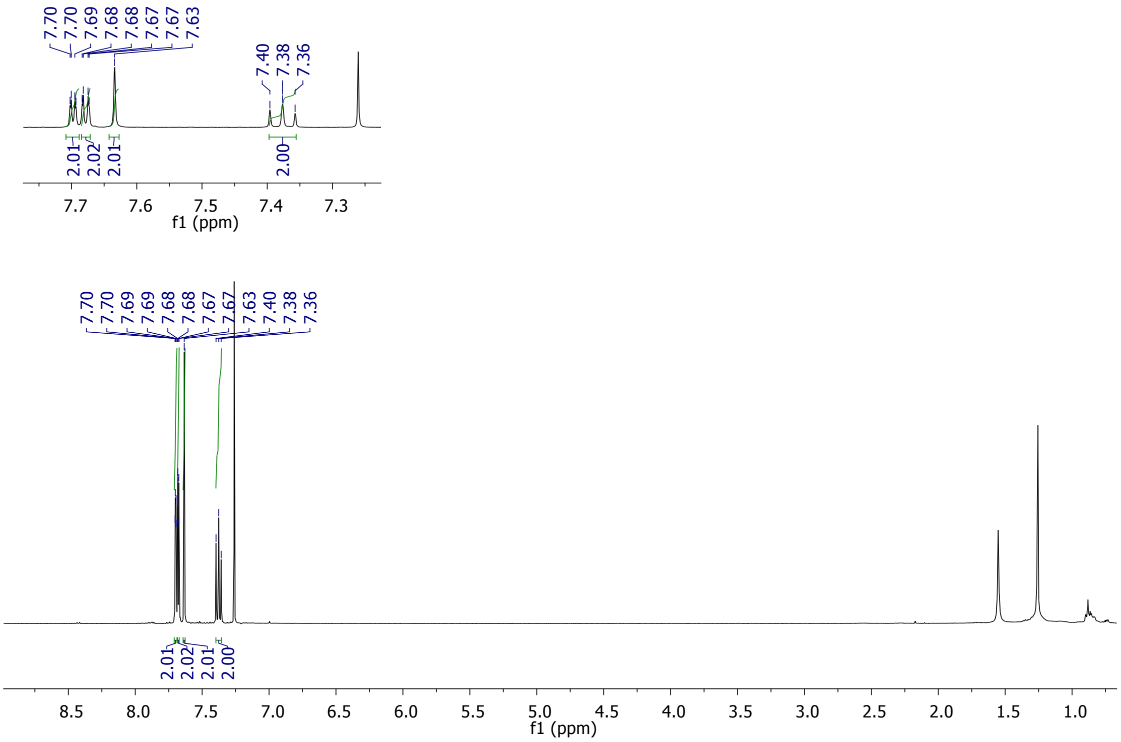

Figure S15. ^{1}H NMR (400 MHz, $CDCl_3$, 298 K) of compound **2**.

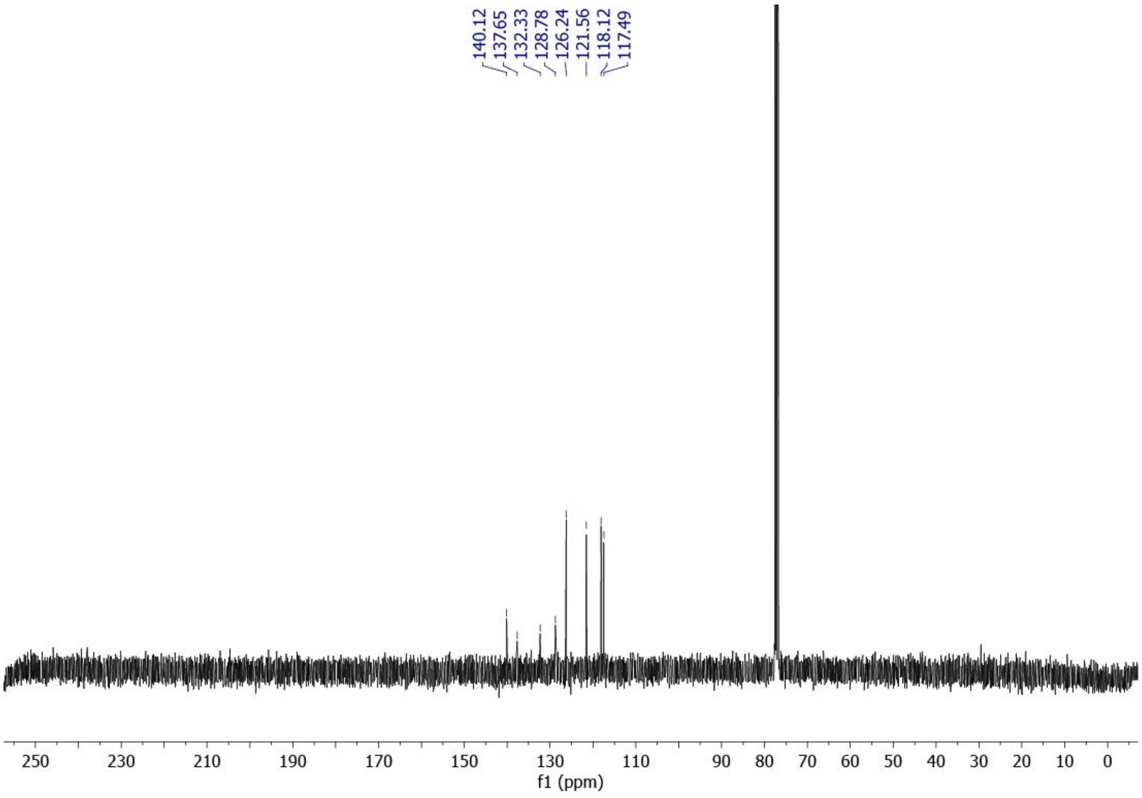

Figure S16. ^{13}C NMR (176 MHz, $CDCl_3$, 298 K) of compound **2**.

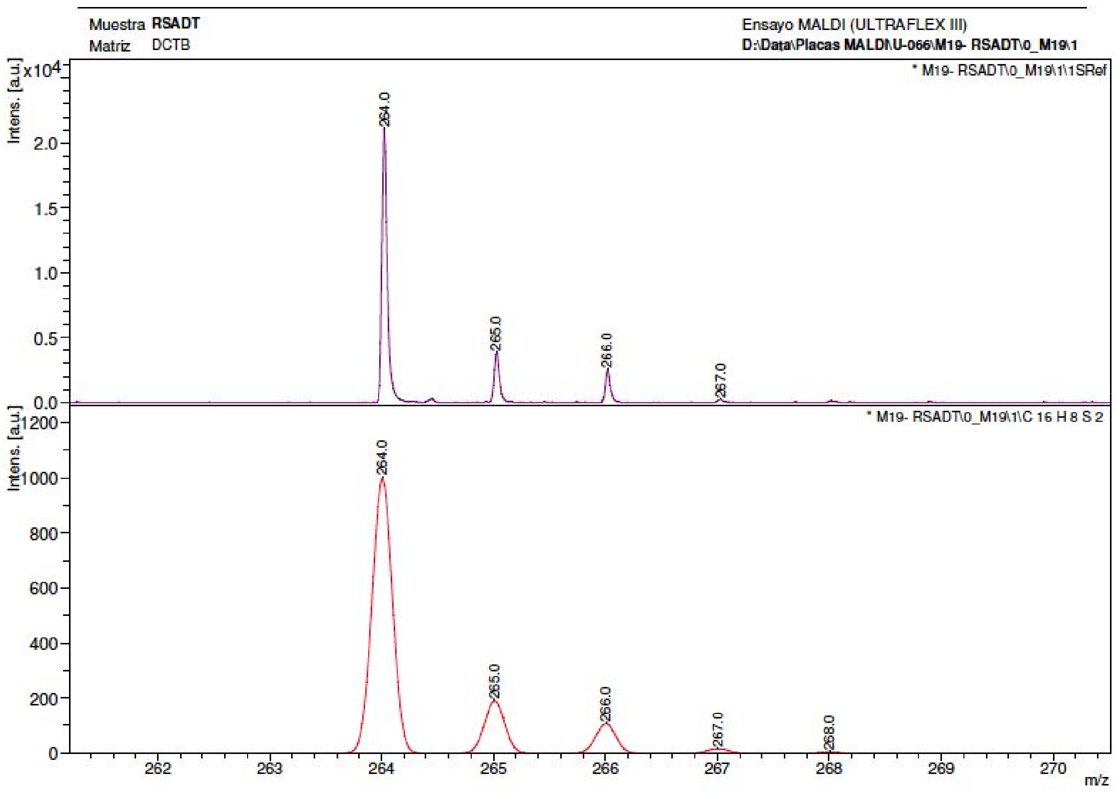

Figure S17. MALDI-TOF mass spectrum of **2**.

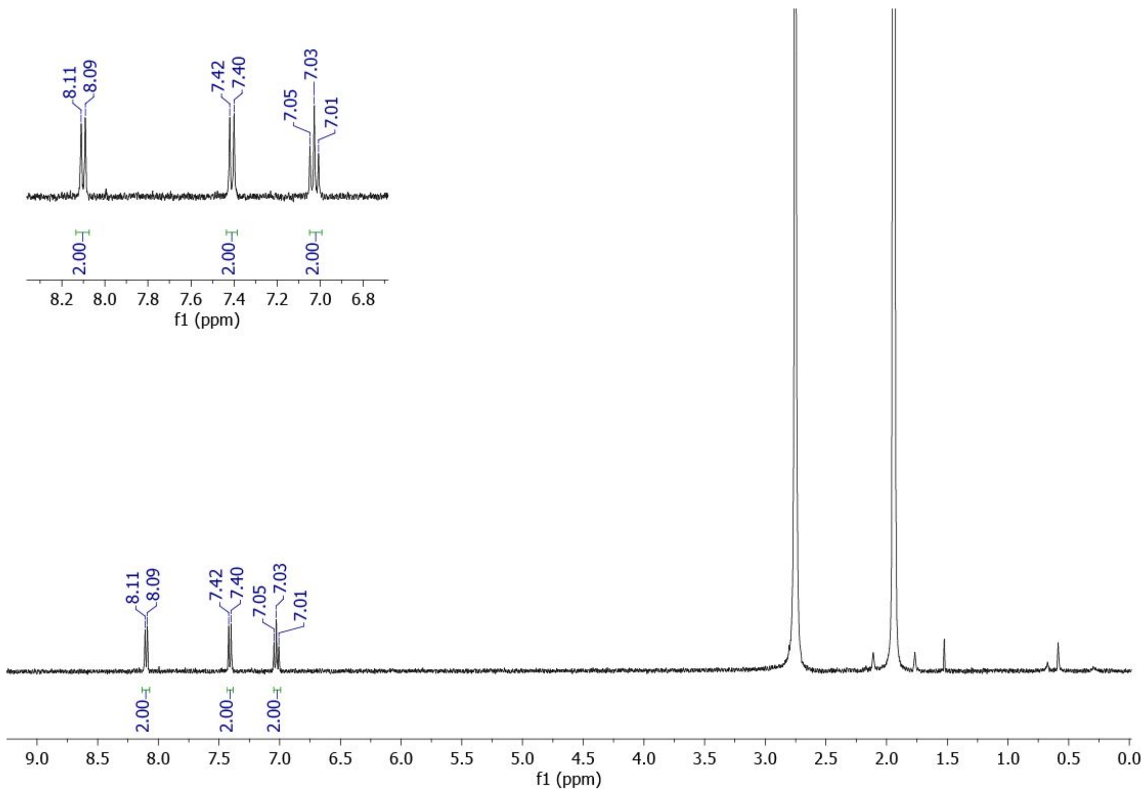

Figure S18. ^{1}H NMR (400 MHz, DMSO-d6, 298 K) of compound **3**.

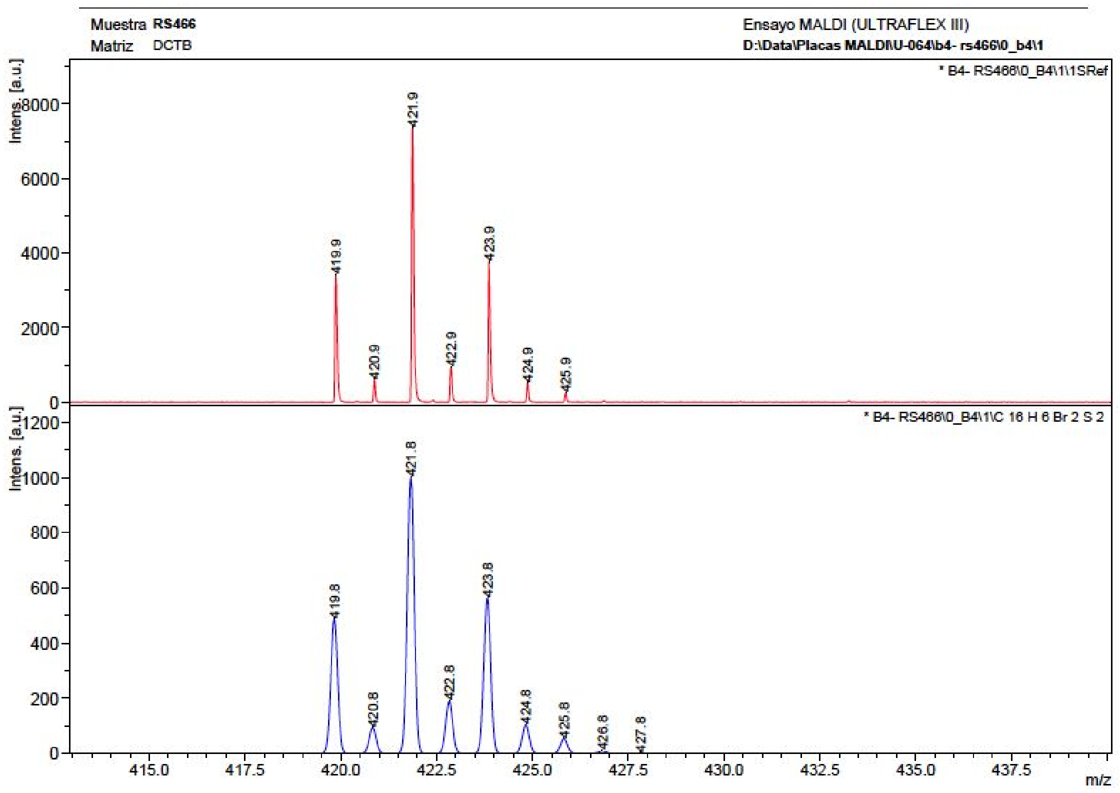

Figure S19. MALDI-TOF mass spectrum of **3**.

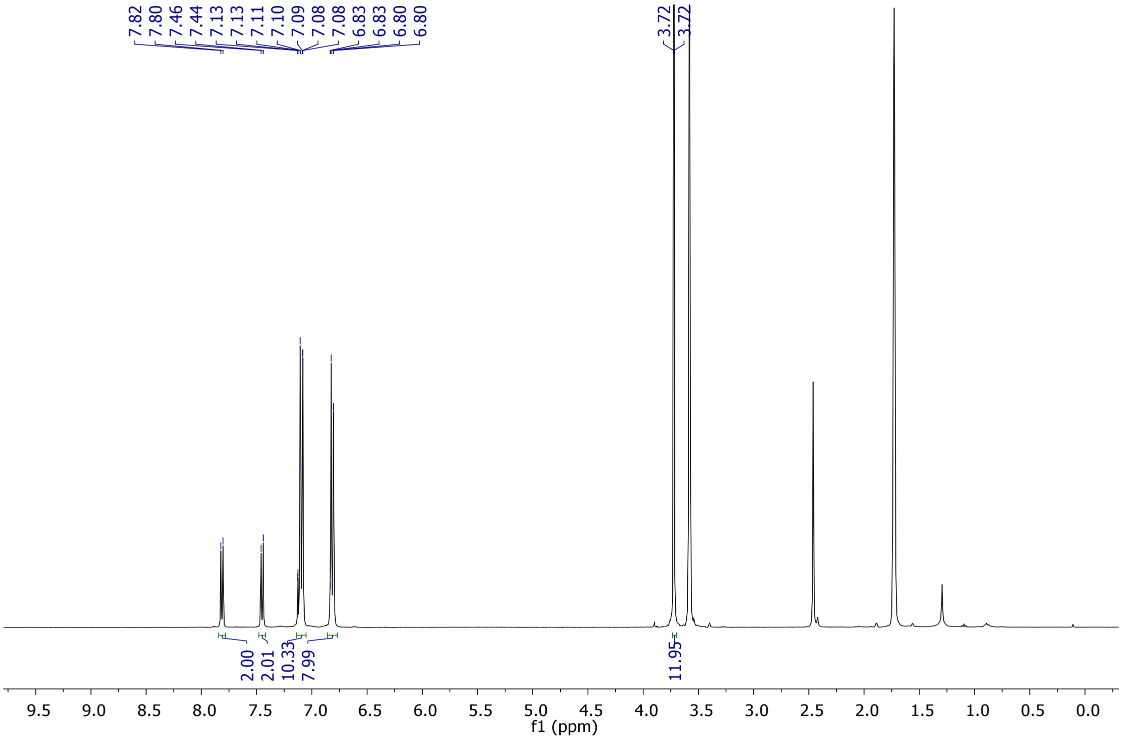

Figure S20. ^{1}H NMR (400 MHz, THF-d^{8}, 298 K) of **ADT-DPA**.

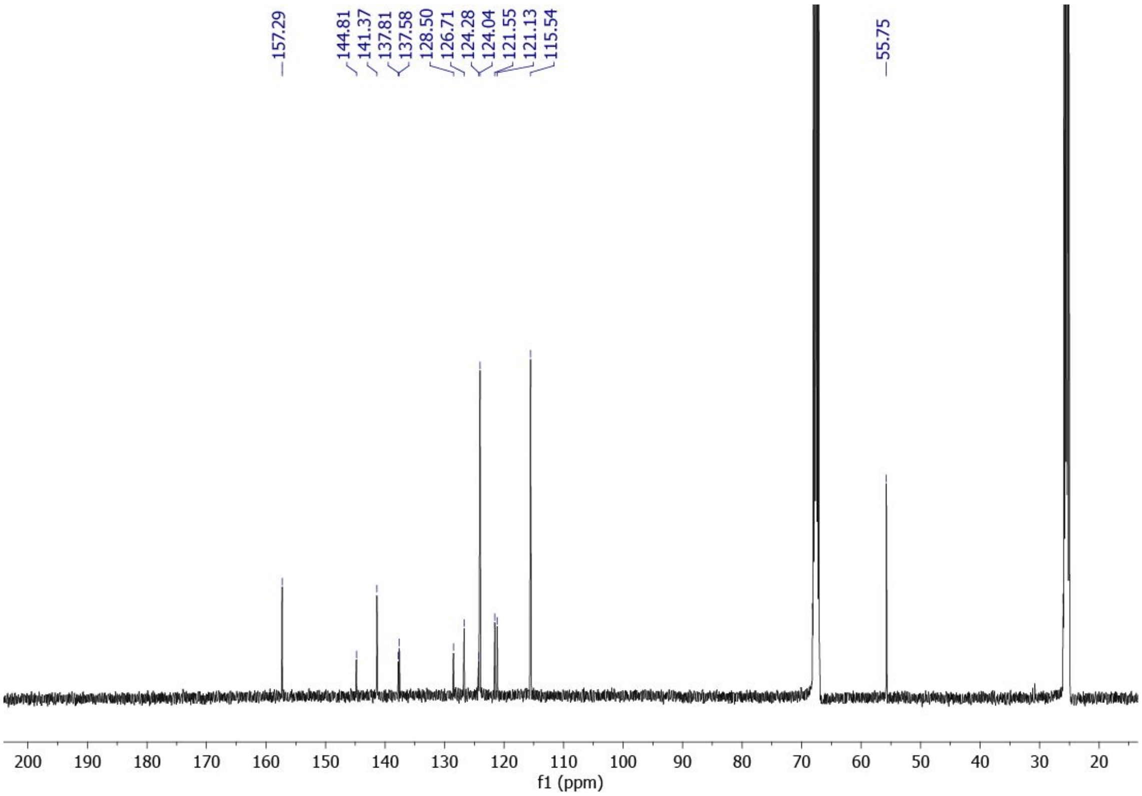

Figure S21. ^{13}C NMR (176 MHz, THF-d^8, 298 K) of **ADT-DPA**.

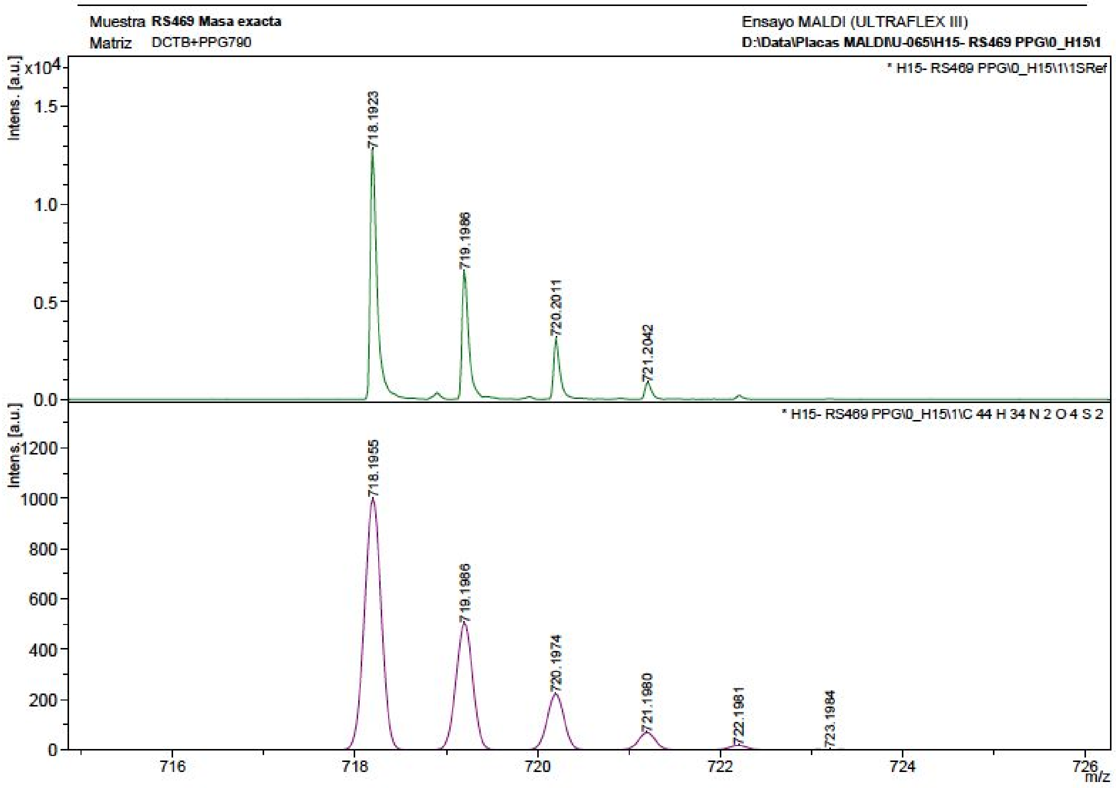

Figure S22. MALDI-TOF mass spectrum of **ADT-DPA**.

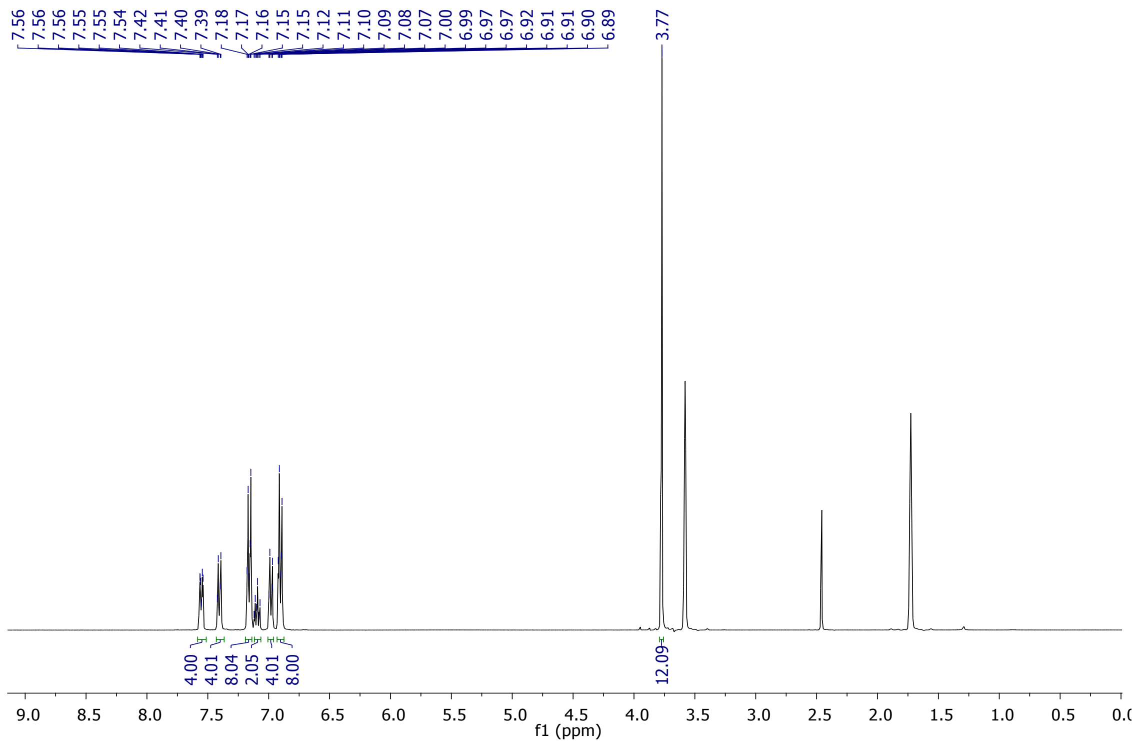

Figure S23. ^{1}H NMR (400 MHz, THF-d^{8}, 298 K) of **ADT-TPA**.

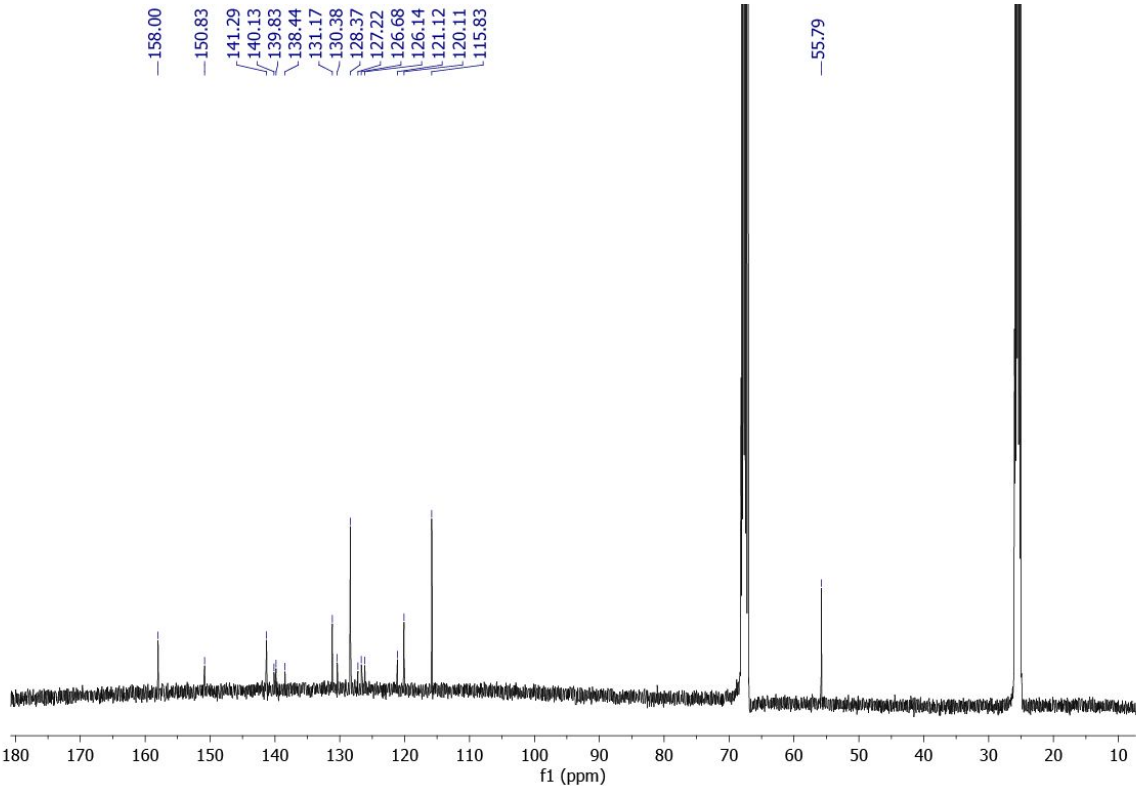

Figure S24. ^{13}C NMR (176 MHz, THF-d^{8}, 298 K) of **ADT-TPA**.

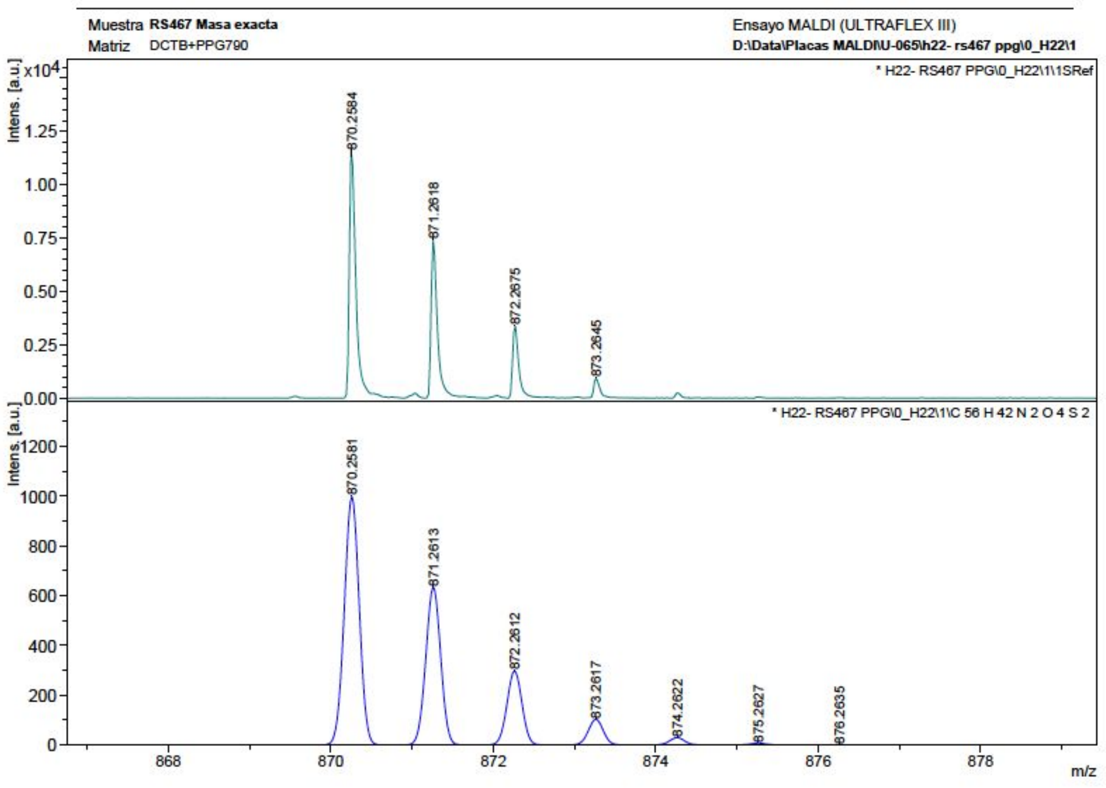

Figure S25. MALDI-TOF mass spectrum of **ADT-TPA**.

7. References

(1) Hill, L. L.; Moore, L. R.; Huang, R.; Craciun, R.; Vincent, A. J.; Dixon, D. A.; Chou, J.; Woltermann, C. J.; Shaughnessy, K. H. Bulky Alkylphosphines with Neopentyl Substituents as Ligands in the Amination of Aryl Bromides and Chlorides. *J. Org. Chem.* **2006**, *71*, 5117−5125.

(2) Rakstys, K.; Abate, A.; Dar, M. I.; Gao, P.; Jankauskas, V.; Jacopin, G.; Kamarauskas, E.; Kazim, S.; Ahmad, S.; Grätzel, M.; Nazeeruddin, M. K. Triazatruxene-Based Hole Transporting Materials for Highly Efficient Perovskite Solar Cells. *J. Am. Chem. Soc.* **2015**, *137*, 16172−16178.

(3) Gaussian16, Revision A.03, Frisch, M. J.; Trucks, G. W.; Schlegel, H. B.; Scuseria, G. E.; Robb, M. A.; Cheeseman, J. R.; Scalmani, G.; Barone, V.; Petersson, G. A.; Nakatsuji, H.; Li, X.; Caricato, M.; Marenich, A. V.; Bloino, J.; Janesko, B. G.; Gomperts, R.; Mennucci, B.; Hratchian, H. P.; Ortiz, J. V.; Izmaylov, A. F.; Sonnenberg, J. L.; Williams-Young, D.; Ding, F.; Lipparini, F.; Egidi, F.; Goings, J.; Peng, B.; Petrone, A.; Henderson, T.; Ranasinghe, D.; Zakrzewski, V. G.; Gao, J.; Rega, N.; Zheng, G.; Liang, W.; Hada, M.; Ehara, M.; Toyota, K.; Fukuda, R.; Hasegawa, J.; Ishida, M.; Nakajima,

T.; Honda, Y.; Kitao, O.; Nakai, H.; Vreven, T.; Throssell, K.; Montgomery Jr., J. A.; Peralta, J. E.; Ogliaro, F.; Bearpark, M. J.; Heyd, J. J.; Brothers, E. N.; Kudin, K. N.; Staroverov, V. N.; Keith, T. A.; Kobayashi, R.; Normand, J.; Raghavachari, K.; Rendell, A. P.; Burant, J. C.; Iyengar, S. S.; Tomasi, J.; Cossi, M.; Millam, J. M.; Klene, M.; Adamo, C.; Cammi, R.; Ochterski, J. W.; Martin, R. L.; Morokuma, K.; Farkas, O.; Foresman, J. B.; Fox, D. J. Gaussian Inc. Wallingford CT, **2016**.

(4) a) Lee, C.; Yang, W.; Parr, R. G. Development of the Colle-Salvetti correlation-energy formula into a functional of the electron density. *Phys. Rev. B* **1988**, *37*, 785−789; b) Becke, A. D. Density-Functional Thermochemistry. III. The Role of Exact Exchange. *J. Chem. Phys.* **1993**, *98*, 5648−5652.

(5) Francl, M. M.; Pietro, W. J.; Hehre, W. J.; Binkley, J. S.; Gordon, M. S.; Defrees, D. J.; Pople, J. A. Self-Consistent Molecular Orbital Methods. XXIII. A Polarization-Type Basis Set for Second-Row Elements. *J. Chem. Phys.* **1982**, *77*, 3654−3665.

(6) a) Tomasi, J.; Persico, M. Molecular Interactions in Solution: An Overview of Methods Based on Continuous Distributions of the Solvent. *Chem. Rev.* **1994**, *94*, 2027–2094; b) Tomasi, J.; Mennucci, B.; Cammi, R. Quantum Mechanical Continuum Solvation Models. *Chem. Rev.* **2005**, *105*, 2999–3093; c) Cramer, C. S.; Truhlar, D. G.; in: Solvent Effects and Chemical Reactivity (Eds.: O. Tapia, J. Bertrán), Kluwer, Dordrecht, The Netherlands, **1996**, p. 1–80.

(7) a) Casida, M. E.; Jamorski, C.; Casida, K. C.; Salahub, D. R. Molecular Excitation Energies to High-Lying Bound States from Time-Dependent Density-Functional Response Theory: Characterization and Correction of the Time-Dependent Local Density Approximation Ionization Threshold. *J. Chem. Phys.* **1998**, *108*, 4439–4449; b) Jamorski, C.; Casida, M. E.; Salahub, D. R. Dynamic Polarizabilities and Excitation Spectra from a Molecular Implementation of Time-Dependent Density-Functional Response Theory: N_2 as a Case Study. *J. Chem. Phys.* **1996**, *104*, 5134–5147; c) Petersilka, M.; Gossmann, U. J.; Gross, E. K. U. Excitation Energies from Time-Dependent Density-Functional Theory. *Phys. Rev. Lett.* **1996**, *76*, 1212–1215.

(8) From http://www.chemcraftprog.com
